# Humidity-Triggered Reversible 0–1D Phase Transition in Hybrid Antimony Halides

**DOI:** 10.3390/nano15060442

**Published:** 2025-03-14

**Authors:** Yi Liu, Jiahua Luo, Abdusalam Ablez, Jinmei Liu, Nianhao Wang, Haowei Lin, Zeping Wang, Xiaoying Huang

**Affiliations:** 1 College of Chemistry, Fuzhou University, Fuzhou 350108, China; yiliu@fjirsm.ac.cn (Y.L.); luojiahua@fjirsm.ac.cn (J.L.); abdslm@fjirsm.ac.cn (A.A.); liujinmei@fjirsm.ac.cn (J.L.); wangnianhao@fjirsm.ac.cn (N.W.); 2 State Key Laboratory of Structural Chemistry, Fujian Institute of Research on the Structure of Matter, Chinese Academy of Sciences, Fuzhou 350002, China; linhw@fjirsm.ac.cn; 3 Fujian College, University of Chinese Academy of Sciences, Fuzhou 350002, China

**Keywords:** structural dimensionality change, inorganic–organic hybrid metal halides, humidity sensitive material, fluorescent sensor, trace water detection

## Abstract

Stimulus-responsive inorganic–organic hybrid metal halides (IOMHs) have shown great potential in applications such as sensing and anti-counterfeiting. IOMHs can undergo a variety of structural changes when triggered by humidity; however, relevant reports of structural dimensionality change from zero dimension (0D) to one dimension (1D) are rare. This study investigates the synthesis, structure, and properties of two antimony-based IOMHs, namely 0D-(Mp)_3_SbCl_6_·MeCN and 1D-(Mp)_2_SbCl_5_ (Mp = protonated morpholine; MeCN = acetonitrile). Photophysical characterizations show that (Mp)_3_SbCl_6_·MeCN, when being excited at 375 nm, exhibits typical self-trapped exciton triplet state broad-band emission, with a peak at 620 nm and a quantum yield as high as 75.06%. Under humid conditions, the 0D structure of (Mp)_3_SbCl_6_·MeCN undergoes a phase transition, leading to the 1D structure of (Mp)_2_SbCl_5_. This transition is accompanied by fluorescence quenching. X-ray powder diffraction, Raman spectroscopy, and thermogravimetric analysis confirm the phase transition process and its reversibility. Based on the high contrast of fluorescence before and after phase transition, (Mp)_3_SbCl_6_·MeCN is demonstrated as an ideal material for fluorescence water sensing, capable of detecting trace amounts of water in tetrahydrofuran with a detection limit of 0.2% *v*/*v*.

## 1. Introduction

Inorganic–organic hybrid metal halides (IOMHs) have garnered substantial attention owing to their tunable electronic structures, optoelectronic properties, and adaptable organic–inorganic components or dimensionality [1,2,3,4,5,6,7,8,9]. These materials adopt the general formula of *A_m_MX_n_* (*A* = cation; *M* = metal ion; *X* = halide ion) [10]. Their structure typically comprises positively charged organic *A*-site cations and negatively charged [*MX_n_*]*^l^*^−^ inorganic frameworks linked by ionic bonds. When organic cations are bulky or display dynamic behavior such as rotational motion, their reduced charge density leads to weaker ionic interactions compared to those of conventional inorganic salts like NaCl. This weaker bonding may serve to make the lattice softer [11]. Consequently, IOMHs can undergo structural changes, such as the distortion of the [*MX_n_*]*^l^*^−^ structure, or even phase transition, when being subjected to external stimuli such as pressure, temperature, and humidity [12,13,14,15]. These structural changes lead to corresponding changes in properties, such as fluorescence [12,14,16,17,18,19].

A large number of studies have reported that humidity changes trigger diverse structural transformations in IOHMs, resulting in significant changes in their fluorescence properties. These changes manifest as highly sensitive fluorescence responses which enable IOHMs to exhibit excellent performance in moisture detection. For instance, under the influence of humidity, Mn-based and Cu-based IOMHs experienced changes in coordination numbers [15,20,21,22,23,24,25,26,27,28,29,30,31,32,33]. Guo et al. reported that the non-emissive bimetallic complex (EtPh_3_P)CuBr_2_ and the yellow-emitting trimetallic complex (EtPh_3_P)_2_Cu_2_Br_4_ could be transformed into the orange-yellow-emitting hexacoordinated (EtPh_3_P)_2_Cu_4_Br_6_ upon water exposure [33]. Zang et al. reported that the green-emitting tetracoordinated (C_6_N_2_H_16_)MnBr_4_ (C_6_N_2_H_16_ = protonated trans-2,5-dimethylpiperazine) could take up water molecules and become the non-emissive hexacoordinated (C_6_N_2_H_16_)MnBr_4_(H_2_O)_2_ [30]. The insertion and removal of water molecules significantly affected the hydrogen bonding, resulting in changes to the distortion levels of [*MX_n_*]*^l^*^−^, which in turn caused corresponding shifts in fluorescence emission. Lei et al. reported that the insertion of water molecules into the red-emitting *β*-[DHEP]SbCl_5_ (DHEP = protonated 1,4-Di(2-hydroxyethyl)piperazine) transforms it into the yellow-emitting *β*-[DHEP]SbCl_5_·(H_2_O)_2_ [29]. The insertion of water molecules increased the hydrogen bonding between the organic and inorganic components, and the more compact environment around the [SbCl_5_]^2−^ unit reduced the distortion level, causing the emission wavelength to shift to higher energy. Humidity-triggered phase transitions in IOMHs led to a significant reduction in the metal–metal distance in the [*MX_n_*]^m−^ structure after phase change, resulting in fluorescence transformation effects. Wang et al. reported that the orange-yellow-emitting [Di-MA]_2_SbCl_5_·MeCN (Di-MA = dimethylammonium), the orange-yellow-emitting [Tetra-MA]_2_SbCl_5_·DMF (Tetra-MA = tetramethylammonium), and the orange-emitting [DEDMA]_2_SbCl_5_ (DEDMA = diethyldimethylammonium) could undergo phase transitions in environments with humidity above 73% relative humidity (RH), transforming into non-emissive [Di-MA]_3_Sb_2_Cl_9_, [Tetra-MA]_3_Sb_2_Cl_9_, and [DEDMA]_3_Sb_2_Cl_9_, respectively [32]. Under the influence of humidity, IOMHs even underwent dimensional changes. Gao et al. reported that in the 0D structure of (*S*-/*R*-MBABr)_2_[MnBr_4_] (MBA = methylbenzylammonium), the asymmetric hydrogen bonding and unsaturated coordination environment of the manganese atoms are sensitive to humidity, leading to a phase transition into the 1D structure of (*S*-/*R*-MBA)[MnBr_3_(EtOH)] [28]. Sharma et al. reported that with increasing moisture, the excess MABr in the 0D MA_4_PbBr_6_ (MA = methylammonium ion) undergoes continuous dissolution and crystallization, causing a structural transition to the 3D MAPbBr_3_ [31]. Due to their high sensitivity, ease of use, and low cost, IOHMs are ideal materials for detecting trace amounts of water in organic solvents. Despite the numerous studies on humidity-induced structural changes, research on their application for detecting trace water in organic solvents is still limited. Under humidity, the structure of 0D antimony-based hybrid metal halides often undergoes a phase transition, with the anion part transforming into a 0D dimeric [*M*_2_*X*_9_]^3−^ unit [32,34]. By contrast, to our knowledge, the structural transition from 0D to 1D has not been documented for antimony-based IOHMs.

In this study, two hybrid antimony-based halide crystals, (Mp)_3_SbCl_6_·MeCN and (Mp)_2_SbCl_5_ (Mp = protonated morpholine), were successfully synthesized using a room-temperature solution method and diffusion method, respectively. The inclusion of acetonitrile molecules in (Mp)_3_SbCl_6_·MeCN enhances supramolecular interactions, which helps suppress non-radiative transitions, resulting in bright emission with a quantum yield up to 75.06%. Under the influence of humidity, the 0D (Mp)_3_SbCl_6_·MeCN transforms into the 1D (Mp)_2_SbCl_5_. The sudden reduction in the Sb-Sb distance leads to fluorescence quenching, exhibiting a clear fluorescence switching process before and after the structural transition. The emission can be reactivated by heating and re-injecting acetonitrile. X-ray powder diffraction, Raman spectroscopy, and thermogravimetric analysis confirm this reversible phenomenon. The high contrast of fluorescence behavior during the structural transition makes (Mp)_3_SbCl_6_·MeCN an ideal material for fluorescence-based water sensing. This study shows that (Mp)_3_SbCl_6_·MeCN can detect trace amount of water in tetrahydrofuran, with a detection limit of 0.2% *v*/*v*.

## 2. Results and Discussion

### 2.1. Description of the Structures

**Figure 1:**
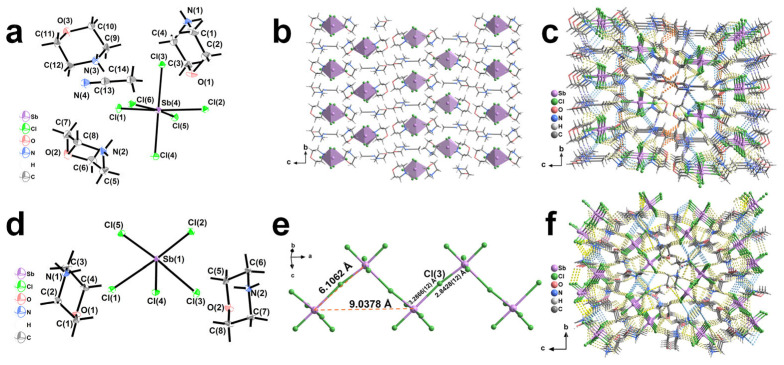
(**a**) *ORTEP* drawing (50% ellipsoid probability) of the asymmetric unit of compound (Mp)_3_SbCl_6_·MeCN. (**b**) View of (Mp)_3_SbCl_6_·MeCN along the *a*-axis, showing the alternating arrangements of [SbCl_6_]^3−^ octahedra/MeCN layers and organic Mp^+^ double layers along *c*-axis. (**c**) The stacking diagram of (Mp)_3_SbCl_6_·MeCN viewed along the *a*-axis. Yellow dashed lines represent C-H···Cl hydrogen bonds, blue dashed lines represent N-H···Cl hydrogen bonds, purple dashed lines represent C-H···N hydrogen bonds, and orange dashed lines represent C-H···O bonds. (**d**) *ORTEP* drawing (50% ellipsoid probability) of the asymmetric unit of compound (Mp)_2_SbCl_5_. (**e**) The Z-shaped one-dimensional chain of (Mp)_2_SbCl_5_. (**f**) The stacking diagram of (Mp)_2_SbCl_5_ viewed along the *a*-axis. Yellow dashed lines represent C-H···Cl hydrogen bonds, blue dashed lines represent N-H···Cl bonds, purple dashed lines represent C-H···N hydrogen bonds, and pink dashed lines represent N-H···O hydrogen bonds.

Single-crystal X-ray diffraction (SCXRD) analysis shows that compound (Mp)_3_SbCl_6_·MeCN crystallizes in the orthorhombic *P*2_1_2_1_2_1_ space group, with unit cell parameters of *a* = 8.6622(3) Å, *b* = 8.8904(3) Å, *c* = 33.1475(11) Å, *V* = 2552(15) Å^3^, and *Z* = 4 (for detailed crystallographic data, see Table S1). The asymmetric unit contains one [SbCl_6_]^3−^ unit, three Mp^+^ cations, and one guest molecule of MeCN (Figure 1a). The Sb-Cl bond lengths range from 2.4531(11) to 3.0518(11) Å, the Sb···Sb distance is 8.3921 Å, and the Cl-Sb-Cl bond angles range from 86.81(3)° to 175.86(4)° (Table S2). Similarly to previously reported 0D compounds with [SbCl_6_]^3−^ anionic components, (Mp)_3_SbCl_6_·MeCN features the [SbCl_6_]^3−^ octahedra layers along the *ab*-plane, which are perfectly separated by Mp^+^ organic layers (Figure 1b) [35]. The distance between adjacent [SbCl_6_]^3−^ units is 8. 3921 Å (Figure S1), implying that the interactions between the anions are relatively weak [36,37]. This unique low-dimensional structure, with the inorganic components fully separated by the organic components, endow (Mp)_3_SbCl_6_·MeCN with an ideal low-dimensional metal halide [38]. Additionally, the structure contains numerous weak interactions, such as C-H···Cl, C-H···O, and N-H···Cl non-classic hydrogen bonds (Table S3). The anion [SbCl_6_]^3−^ units are interacted with the Mp^+^ cations through hydrogen bonds of C-H···Cl and N-H···Cl to form a two-dimensional anionic layered structure (Figure S2). Further C-H···O and C-H···N interactions between layers result in a three-dimensional supramolecular framework (Figure 1c and Figure S3). The existence of MeCN contributes more hydrogen bonds, such as C(14)-H(14B)···Cl(2) and all C-H···N hydrogen bonds (Figure S4).

(Mp)_2_SbCl_5_ also crystallizes in the orthorhombic *P*2_1_2_1_2_1_ space group, with unit cell parameters of *a* = 9.0378(4) Å, *b* = 10.1349(4) Å, *c* = 17.8262(6) Å, *V* = 1632.83(11) Å^3^, and *Z* = 4 (for detailed crystallographic data, see Table S1). The asymmetric unit contains one [SbCl_5_]^2−^ anion and two Mp^+^ cations (Figure 1d). The Sb-Cl bond lengths range from 2.44133(12) to 2.8929(11) Å, while a slightly longer secondary Sb(1)-Cl(3) bond, with a length of 3.2866(12) Å, is present, forming a distorted [SbCl_6_] octahedron. The Sb···Sb distance is 6.1062(5) Å (Figure 1e), and the Cl-Sb-Cl bond angles range from 84.75° to 177.17° (Table S2). Adjacent [SbCl_6_] octahedra forms a Z-shaped one-dimensional chain along the *a*-axis by sharing the Cl(3) atom through a self-assembly process (Figure 1e). Similarly, the rich hydrogen bonding interactions, such as C-H···Cl, C-O···H, and N-H···Cl hydrogen bonds in (Mp)_2_SbCl_5_ contribute to the formation of a three-dimensional supramolecular structure (Figure 1f, Figures S5 and S6 and Table S3).

X-ray powder diffraction (PXRD) characterization confirms that both (Mp)_3_ SbCl_6_·MeCN and (Mp)_2_SbCl_5_ are phase-pure (Figure S7).

### 2.2. Photoluminescence Performance Description

**Figure 2:**
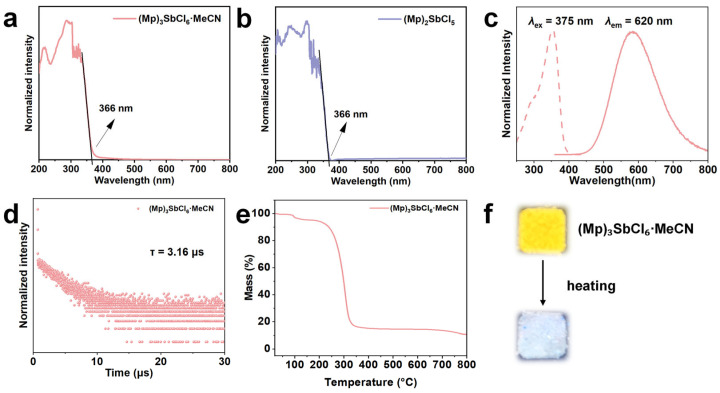
Absorption spectra of (Mp)_3_SbCl_6_·MeCN (**a**) and (Mp)_2_SbCl_5_ (**b**). (**c**) Photoluminescence excitation (PLE) and PL spectra of (Mp)_3_SbCl_6_·MeCN. (**d**) Fluorescence lifetime of (Mp)_3_SbCl_6_·MeCN. (**e**) The TGA curve of (Mp)_3_SbCl_6_·MeCN. (**f**) Fluorescence changes in (Mp)_3_SbCl_6_·MeCN before and after heat treatment.

(Mp)_3_SbCl_6_·MeCN and (Mp)_2_SbCl_5_ single crystals are both transparent and colorless plate-like crystals under ambient light. Under a 365 nm UV lamp, (Mp)_3_SbCl_6_·MeCN exhibits bright yellow fluorescence, while (Mp)_2_SbCl_5_ is non-emissive at room temperature. The photophysical properties of (Mp)_3_SbCl_6_·MeCN were systematically characterized using steady-state photoluminescence (PL) spectroscopy and time-resolved PL spectroscopy. As shown, both (Mp)_3_SbCl_6_·MeCN and (Mp)_2_SbCl_5_ exhibit a sharp absorption edge around 366 nm (Figure 2a,b).

The PL excitation spectrum of (Mp)_3_SbCl_6_·MeCN displays a broadband feature, with the main peak at 375 nm and a weak shoulder excitation peak at 298 nm. It closely matches the absorption edge, indicating that the PL emission originates from the intrinsic bulk phase of the crystal rather than surface defects [39]. Under 375 nm UV excitation, exhibits a single broad emission spectrum (430–800 nm), peaking at 620 nm (Figure 2c). The full width at half-maximum (FWHM) is 144 nm, and the Stokes shift is 245 nm. The broad emission spectrum of (Mp)_3_SbCl_6_·MeCN shows an ideal Gaussian distribution, indicating a single optical center with minimal excitation-state energy [35]. The PL quantum yield (PLQY) is 75.06%. The calculated Commission Internationale de l’Éclairage (CIE) chromaticity coordinates are (0.47, 0.49), corresponding to the yellow light typically found in 0D hybrid antimony halides (Figure S8). To elucidate the physical process behind the broadband emission, time-resolved PL spectra of the emission peak were measured at 300 K. As shown in Figure 2b, the lifetime is 3.16 μs. Based on the large FWHM, significant Stokes shift, and long lifetime, the broad-band emission of (Mp)_3_SbCl_6_·MeCN can be attributed to the triplet-state STE radiative recombination induced by strong electron–phonon coupling in a deformable, soft lattice [40,41,42,43].

Thermogravimetric analysis (TGA) shows that (Mp)_3_SbCl_6_·MeCN exhibits a weight loss of 4.63% around 100 °C, which is close to the theoretical MeCN desorption amount (6.41%) (Figure 2e). The experimental value is slightly lower, possibly due to some MeCN volatilizing in the environment before testing. The compound then completely decomposes at around 340 °C. A noticeable fluorescent quenching was observed upon heating for (Mp)_3_SbCl_6_·MeCN, with no recovery after cooling. This is likely caused by the desorption of MeCN molecules from the compound. Therefore, it can be concluded that MeCN molecules are key to the luminescence of (Mp)_3_SbCl_6_·MeCN (Figure 2f). (Mp)_2_SbCl_5_ begins to thermally decompose around 210 °C and completely decomposes at approximately 340 °C (Figure S9).

**Figure 3:**
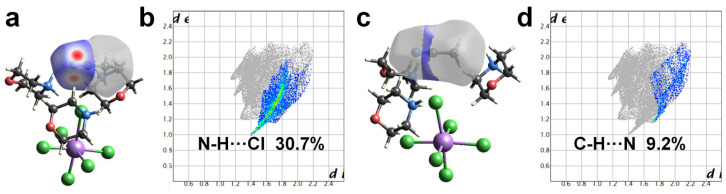
Hirshfeld surface analysis of N-H···Cl in (Mp)_3_SbCl_6_·MeCN (**a**) and its 2D fingerprint plot (**b**). Hirshfeld surface analysis of C-H···N in (Mp)_3_SbCl_6_·MeCN (**c**) and its 2D fingerprint plot (**d**).

There are several reasons why the incorporation of solvent molecules into the structure causes changes in fluorescence. Firstly, the solvent molecules in the structure make the metal halide units more dispersed, preventing electronic interactions and energy transfer between adjacent metal halide units. As a result, photo-induced excitons become highly localized on isolated metal halide units, exhibiting intrinsic quantum confinement effects and high exciton binding energy [44]. Secondly, the introduction of solvent molecules into the structure may also decrease the void fraction of IOMHs, and it increases their PLQY [33]. Thirdly, the solvent molecules may form strong hydrogen bonds with the inorganic metal anion units, changing the degree of distortion and electronic mobility change and thus affecting their luminescent behavior [3,45]. In (Mp)_3_SbCl_6_·MeCN, abundant hydrogen bonds are formed between MeCN molecules and [SbCl_6_]^3−^ inorganic anions, specifically the C(14)-H(14B)···Cl(2) interaction, which enriches the supramolecular interactions, resulting in high PLQY of (Mp)_3_SbCl_6_·MeCN. To better visualize the hydrogen bonds and supramolecular forces in (Mp)_3_SbCl_6_·MeCN, we conducted a Hirshfeld surface analysis and derived the corresponding 2D fingerprint plots (Figure 3). As shown in Figure 3a,b, the presence of MeCN not only promotes strong hydrogen bonding between organic and inorganic components but also leads to rigid supramolecular structures due to rich supramolecular interactions, which helps to suppress non-radiative transitions [8,46,47,48,49,50].

### 2.3. Mechanism Analysis

**Figure 4:**
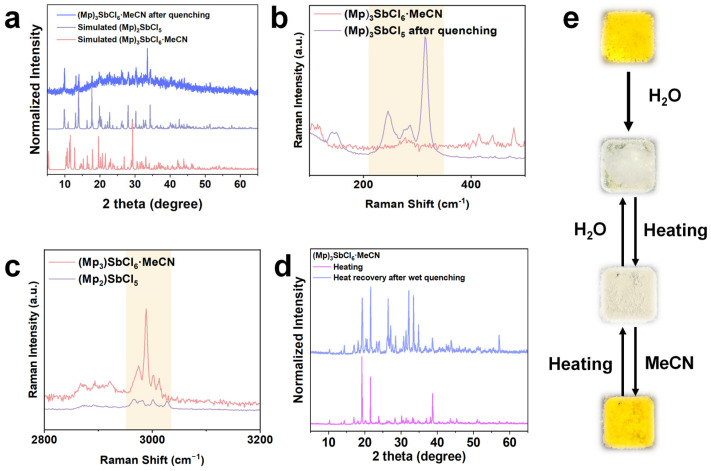
(**a**) PXRD pattern of (Mp)_3_SbCl_6_·MeCN after phase transition triggered by humidity, compared with simulated ones for (Mp)_3_SbCl_6_·MeCN and (Mp)_2_SbCl_5_. (**b**) Raman spectra of (Mp)_3_SbCl_6_·MeCN and quenched samples in the 2500–3200 cm^−1^ range. (**c**) Raman spectra of (Mp)_3_SbCl_6_·MeCN and quenched samples in the 100–500 cm^−1^ range. (**d**) PXRD comparison of quenched material and (Mp)_3_SbCl_6_·MeCN after heating at 100 °C for 1 h during the reversible process. (**e**) Fluorescence changes during the phase transition between (Mp)_3_SbCl_6_·MeCN and (Mp)_2_SbCl_5_ under ultraviolet light.

We observed that the 0D-(Mp)_3_SbCl_6_·MeCN also undergoes fluorescence quenching when exposed to a high-humidity environment. By comparing the PXRD patterns before and after fluorescence quenching, we found that (Mp)_3_SbCl_6_·MeCN undergoes a phase transition under the influence of water. The simulated PXRD pattern of 1D-(Mp)_2_SbCl_5_ was compared with the experimental one of the hydrated derivative of (Mp)_3_SbCl_6_·MeCN (Figure 4a). The PXRD peaks match perfectly, suggesting that under the influence of water, the SbCl_6_ units undergo chemical bond cleavage and reorganization, leading to the formation of 1D [SbCl_5_]*_n_* chain structures. MeCN is stabilized in the crystal structure through hydrogen bonding. When water molecules are adsorbed, they gradually replace MeCN and form hydrogen bonds with chloride ions and metal ions, leading to structural distortion and triggering the transformation into one-dimensional [SbCl_5_]*_n_^n^*^−^ chains [51,52,53]. The infrared spectroscopy proves that the influence of moisture promotes the structural transformation of the compound through the formation and variation of hydrogen bonds (Figure S11). Along with the decreasing Sb···Sb distance from 8.3921 Å to 6.1062 Å, the transition from 0D to 1D induces a ‘concentration quenching’ effect, resulting in fluorescence quenching after the phase transition [36].

Raman spectroscopy was employed to elucidate the structural evolution before and after quenching (Figure S12). In the 200–400 cm^−1^ range, compared to the initial fluorescent 0D-(Mp)_3_SbCl_6_·MeCN, the converted-non fluorescent material exhibits three continuous characteristic peaks at 245 cm^−1^, 286 cm^−1^, and 314 cm^−1^ (Figure 4b). These peaks arise from the phase transition from [SbCl_6_]^3−^ to the [SbCl_5_]*_n_*^2*n*−^ 1D chain. The structural rigidity of the [SbCl_5_]*_n_*^2*n*−^ chain leads to changes in the Sb-Cl stretching vibration modes, as well as the coupling of bending and twisting vibrations of the chain. In the 3000 cm^−1^ region, (Mp)_3_SbCl_5_·MeCN shows significant signals in the 2800–3000 cm^−1^ range, corresponding to the vibration mode of the C≡N group in MeCN (Figure 4c). Due to the Cl···H-N hydrogen bond within the lattice, a red shift is observed. The weakening of the same-region signals in (Mp)_2_SbCl_5_ confirms the desorption of MeCN during the phase transition.

To verify the reversibility of the phase transition, the converted material and (Mp)_3_SbCl_6_·MeCN were heated at 100 °C for 1 h, and their PXRD patterns were compared (Figure 4d). The peak patterns of both are almost identical. Following this, acetonitrile solution was added, which restored the fluorescence of the non-emission powder to orange-yellow emission (Figure 4e).

Based on the above, we propose a possible phase transition mechanism: Stage I, MeCN desorption (corresponding to a TGA weight loss of 4.63%); Stage II, the 0D structure dissociates, with SbCl_6_^3−^ reorganizing into a one-dimensional chain, leading to the phase transition to (Mp)_2_SbCl_5_ and the release of MpCl. The phase transition process is as follows:

(Mp)_3_SbCl_6_·MeCN ⇌ (Mp)_3_SbCl_6_ + MeCN (Mp)_3_SbCl_6_ + *x*H_2_O ⇌ (Mp)_2_SbCl_5_ + MpCl·*x*H_2_O

(Mp)_3_SbCl_6_·MeCN was subjected to humidity quenching and fluorescence recovery under 95% RH for eight cycles. The fluorescence intensity changes were recorded, showing a good fluorescence stability (Figure S13). After eight cycles, the PXRD pattern of the powder was compared with the simulated one, and a good match was observed (Figure S14). This indicates that the repeated exposure to a humid environment and subsequent recovery does not alter its structural integrity or luminescent properties.

### 2.4. Luminescent Water-Sensing

**Figure 5:**
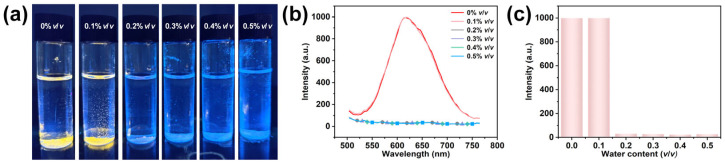
(**a**) (Mp)_3_SbCl_6_·MeCN detection of water content (0~0.5% *v*/*v*) in tetrahydrofuran solvent containing different amounts of water. (**b**) Fluorescence spectra for the compound immersed in tetrahydrofuran solvents containing different amounts of water. (**c**) Comparison of fluorescence intensities for the compound in tetrahydrofuran solvents containing different amounts of water.

Fluorescent water sensors are devices that detect water by observing changes in the optical properties of fluorescent materials (such as fluorescence intensity, wavelength, or lifetime) in the presence of moisture [27,54,55,56]. These sensors are particularly useful for detecting trace amounts of water in organic solvents, offering advantages such as low cost and high sensitivity. We utilized the fluorescence changes induced by the phase transition of (Mp)_3_SbCl_6_·MeCN under the influence of water. By immersing it in tetrahydrofuran with varying water content, (Mp)_3_SbCl_6_·MeCN rapidly quenches within 5 min when the water content reaches 0.2% *v*/*v* (Figure 5a). Fluorescence tests on samples with different water contents show that (Mp)_3_SbCl_6_·MeCN with 0% and 0.1% *v*/*v* water contents maintains good fluorescence, whereas (Mp)_3_SbCl_6_·MeCN with 0.2–0.5% *v*/*v* water contents exhibits fluorescence burst (Figure 5b). As shown in Figure 5c, the contrast in fluorescence intensity also exhibits a high contrast of intense fluorescence before and after the detection limit. With this low detection limit (much lower than that of [HOOCMMIm]_3_SbCl_6_ [12] and (PPZ)_2_SbCl_7_·5H_2_O) [57], (Mp)_3_SbCl_6_·MeCN proves to be an ideal material for trace water analysis.

## 3. Materials and Methods

Morpholine chloride (MpCl, 99%), antimony(III) chloride (SbCl_3_, 99%) and saltpeter (KNO_3_) were purchased from Adamas Reagent Co., Ltd. (Shanghai, China). Methanol (CH_3_OH, AR), acetonitrile (MeCN, AR), and ethyl acetate were purchased from Sinopharm Chemical Reagent Co., Ltd. (Shanghai, China). All reagents were used without further purification.

**Synthesis of compounds (Mp)_3_SbCl_6_·MeCN:** A solution of antimony(III) chloride (SbCl_3_, 0.2282 g, 1 mmol) in 1 mL of acetonitrile (dried over 4 Å molecular sieves) was prepared. This solution was transferred dropwise via a micropipette into a 20 mL glass vial containing MpCl (0.2471 g, 2 mmol). The vial was sealed and heated in a preheated oven at 100 °C for 2 h. After cooling to room temperature, colorless transparent block crystals of (Mp)_3_SbCl*_6_*·MeCN were obtained (yield: 89.92%, calculated based on Sb). EA, anal. calcd. for C_14_H_33_Cl_6_N_4_O_3_Sb of compound (Mp)_3_SbCl_6_·MeCN: C, 26.27; H, 5.19; N, 8.76%; found: C, 27.35; H, 5.49; N, 8.94%.

**Synthesis of compounds (Mp)_2_SbCl_5_:** A mixture of antimony(III) chloride (SbCl_3_, 0.2282 g, 1 mmol) and MpCl (0.2471 g, 2 mmol) was dissolved in 3 mL of methanol. Subsequently, 1 mL of the resulting solution was transferred into a 4 mL glass vial, which was then placed inside a 20 mL glass vial containing 4 mL of ethyl acetate for solvent diffusion at ambient temperature. After 7 days, colorless transparent block crystals of (Mp)_2_SbCl_5_ were obtained (yield: 73.65%, calculated based on Sb). EA, anal. calcd. for C_8_H_20_Cl_5_N_2_O_2_Sb of compound (Mp)_2_SbCl_5_: C, 20.23; H, 4.13; N, 5.90%; found: C, 20.22; H, 4.24; N, 5.89%.

**Single-crystal X-ray diffraction (SCXRD):** Crystals of appropriate size and dimensions were selected under a microscope and then the crystals were fixed at the tip with a glass wire for single-crystal X-ray diffraction (SCXRD) characterization. SCXRD data for all the four title compounds were collected on a Synergy R CCD diffractometer with graphite monochromatic Mo K_α_ radiation (λ = 0.71073 Å). The collection temperature of the title compounds is 100(2) K. Crystallographic data and refinement details for (Mp)_3_SbCl_6_·MeCN and (Mp)_2_SbCl_5_ are shown in Table S1. The structures were solved by direct methods and refined by full-matrix least-squares on F^2^ using the SHELX-2018 program package [58]. Selected bond lengths and angles of compounds (Mp)_3_SbCl_6_·MeCN and (Mp)_2_SbCl_5_ are shown in Table S2. CCDC NO. 2424336 and 2424337 contain the supplementary crystallographic data for this paper. These data can be obtained free of charge from The Cambridge Crystallographic Data Centre via www.ccdc.cam.ac.uk/data_request/cif accessed on 17 February 2025.

**Characterization:** Powder X-ray diffraction (PXRD) patterns were measured on a Rigaku Miniflex-II diffractometer (Japan) by utilizing CuKα radiation (λ = 1.54178 Å) in the angular range of 2θ = 5–65°. Thermogravimetric (TG) analyses were performed on a NETZSCH STA 449F3 (Netzsch, Germany) unit at a heating rate of 10 K·min^−1^ under N2 atmosphere. Photoluminescence excitation (PLE) and photoluminescence (PL) spectra and time-resolved PL spectra and quantum yields of compound (Mp)_3_SbCl_6_·MeCN were recorded on Edinburgh FLS1000 UV/V/NIR (Edinburgh, UK) fluorescence spectrometer. Elemental analysis (EA) was conducted on a German Elementary Vario MICRO instrument (Elementar, Germany). The solid UV diffuse reflectance data were measured and collected using a Shimadzu UV-2600 UV-Vis spectrophotometer (Shimadzu, Japan), with a wavelength range set from 200 to 800 nm. Prior to testing, BaSO_4_ was used as a reference for baseline scanning. The absorption data were calculated using the Kubelka–Munk formula α/S = (1 − R)2/2R, where R is the reflectance coefficient of the sample. Raman spectra were measured on a LabRAM HR confocal Raman spectrometer (Horiba Jobin Yvon, France) with a laser source of 532 nm and a measurement range of 0–4000 cm^−1^.

**Hirshfeld surface analyses:** The molecular interactions of (Mp)_3_SbCl_6_·MeCN and (Mp)_2_SbCl_5_ were analyzed using Hirshfeld surface analysis via the Crystal Explore 17 program [59,60,61,62,63,64]. The Hirshfeld surface of a crystal molecule is constructed by dividing space into different regions. The sum of the electron density of the molecule (the precursor molecule) in these regions, divided by the electron density of spherical atoms, is set to 0.5. The distance from the Hirshfeld surface to the nearest external nucleus is defined as *d_e_*, and the distance to the nearest internal nucleus is defined as *d_i_*. The sum of *d_e_* and *d_i_* is considered as *d_norm_* and is normalized by the van der Waals radius (*r^vdw^*). The red highlights on the Hirshfeld surface represent intermolecular contact distances smaller than the sum of their van der Waals radii, while white highlights indicate contact distances close to the sum of the radii, and blue highlights show longer contact distances. A 2D fingerprint plot is used to summarize intermolecular interactions. It is generated by plotting the distribution of (*d_e_*, *d_i_*) points derived from the Hirshfeld surface [59]. Each point in the 2D fingerprint plot corresponds to a unique (*d_e_*, *d_i_*) pair, and the color indicates the contribution of weak interactions. Red represents the largest contribution, while blue represents the smallest contribution.

## 4. Conclusions

This study investigates the reversible phase transition and fluorescence response of antimony-based hybrid halides triggered by H_2_O. Two homologous antimony-based hybrid halides, (Mp)_3_SbCl_6_·MeCN and (Mp)_2_SbCl_5_, were successfully synthesized using room-temperature solution-based and diffusion methods. This study demonstrated that (Mp)_3_SbCl_6_·MeCN undergoes a dimensionality transformation from 0D to 1D upon water stimulation. The water-induced phase transition leads to a reduction in the Sb-Sb distance, triggering a concentration quenching phenomenon that ultimately results in fluorescence quenching. This reversible phase transition process was confirmed through characterization techniques such as PXRD, Raman spectroscopy, and thermogravimetric analysis. Furthermore, (Mp)_3_SbCl_6_·MeCN demonstrates excellent fluorescence response characteristics in the presence of water, making it a highly sensitive fluorescence water sensor capable of detecting trace amounts of water in tetrahydrofuran, with a detection limit of 0.2% *v*/*v*. This study not only provides new insights into humidity-induced phase transitions but also highlights the potential of antimony-based hybrid halides in sensor applications

## Data Availability

The original contributions presented in this study are included in the article/Supplementary Materials. Further inquiries can be directed to the corresponding authors.

## Supplementary Materials

The following supporting information can be downloaded at https://www.mdpi.com/article/10.3390/nano15060442/s1: Table S1. Reported studies on the structure and properties of (Mp)_3_SbCl_6_·MeCN and (Mp)_2_SbCl compounds; Table S2. Selected bond length (Å) and bond angle (°) for (Mp)_3_SbCl_6_·MeCN and (Mp)_2_SbCl;; Table S3. Hydrogen bond data for (Mp)_3_SbCl_6_·MeCN and (Mp)_2_SbCl_5_; Figure S1. Sb···Sb distance in the anion part of (Mp)_3_SbCl_6_·MeCN; Figure S2. Hydrogen bonds in the [SbCl_6_]^3−^anion of (Mp)_3_SbCl_6_·MeCN; Figure S3. (a) (Mp)_3_SbCl_6_·MeCN is connected by C-H···Cl and N-H···Cl along the a-axis direction to form a two-dimensional layered structure. Yellow dotted lines indicate hydrogen bonds. (b) The three-dimensional supramolecular structure is formed by C-H···N (orange dotted line) and C-H···O (blue dotted line) hydrogen bonds along the b-axis direction; Figure S4. Hydrogen bonds connected with MeCN in (Mp)_3_SbCl_6_·MeCN; Figure S5. Hydrogen bonds in the [SbCl_6_]^3−^anion of (Mp)_2_SbCl_5;_ Figure S6. (a) (Mp)_2_SbCl_5_ is connected by C-H···Cl and N-H···Cl along the a-axis direction to form a two-dimensional layered structure. Yellow dotted lines indicate hydrogen bonds. (b) The three-dimensional supramolecular structure is formed by N-H···O (pink dotted line) hydrogen bonds along the b-axis direction; Figure S7. PXRD patterns of (Mp)_3_SbCl_6_·MeCN (a) and (Mp)_2_SbCl_5_ (b); Figure S8. CIE chromaticity coordinates corresponding to (Mp)_3_SbCl_6_·MeCN. The illustration shows (Mp)_3_SbCl_6_·MeCN emitting orange-yellow fluorescence under a 365 nm ultraviolet lamp; Figure S9. Thermogravimetric (TG) curve of (Mp)_2_SbCl_5_; Figure S10. (a) Hirshfeld surface analysis of (Mp)_3_SbCl_6_·MeCN. (b) Two-dimensional fingerprint plot of (Mp)_3_SbCl_6_·MeCN. (c) Hirshfeld surface analysis of the [SbCl_6_]^3−^ anion part of (Mp)_3_SbCl_6_·MeCN. (d) Two-dimensional fingerprint plot of the [SbCl_6_]^3−^ anion part of (Mp)_3_SbCl_6_·MeCN. (e) Hirshfeld surface analysis of (Mp)_2_SbCl_5_. (f) Two-dimensional fingerprint plot of (Mp)_2_SbCl_5_. (g) Hirshfeld surface analysis of the [SbCl_5_]^2−^ anion part of (Mp)_2_SbCl_5_. (h) Two-dimensional fingerprint plot of the [SbCl_5_]^2−^ anion part of (Mp)_2_SbCl_5_; Figure S11. Infrared spectra of (Mp)_3_SbCl_6_·MeCN before and after humidity quenching; Figure S12. Full Raman spectra of (Mp)_3_SbCl_6_·MeCN and (Mp)_2_SbCl_5_; Figure S13. Fluorescence cycling of (Mp)_3_SbCl_6_·MeCN; Figure S14. The experimental PXRD pattern of (Mp)_3_SbCl_6_·MeCN after 8 cycles of humidity quenching and fluorescence recovery compared with the simulated one from the single crystal X-ray data of (Mp)_3_SbCl_6_·MeCN.

## References

[1] Jin J.-C., Lin Y.-P., Chen D.-Y., Lin B.-Y., Zhuang T.-H., Ma W., Gong L.-K., Du K.-Z., Jiang J., Huang X.-Y. (2021). X-ray scintillation and photoluminescence of isomorphic ionic bismuth halides with [Amim]^+^ or [Ammim]^+^ cations. Inorg. Chem. Front..

[2] Lin H.-W., Ablez A., Deng Z.-H., Chen Z.-H., Peng Y.-C., Wang Z.-P., Du K.-Z., Huang X.-Y. (2024). A ligand-incorporating strategy towards single-component white light in ionic zero-dimensional indium chlorides. J. Mater. Chem. C.

[3] Peng Y.-C., Jin J.-C., Zhou S.-H., Lin H.-W., Huang D.-D., Deng Z.-H., Dong Y., Xu H.-J., Du K.-Z., Wang Z.-P. (2024). Regulating photoluminescence through single-crystal-to-single-crystal transformation of solvent-containing zero-dimensional hybrid metal halide isomers. Chem. Eng. J..

[4] Zang Z.G., Liang D.H., Shi Y.R., Liu Z.Y., Li R., Qaid S.M.H., Cai W.S. (2024). Ethanol-Induced Reversible Phase Transition in Antimony Halides for Morse Code Anti-Counterfeiting and Optical Logic Gates. Laser Photonics Rev..

[5] Zhang Z.-Z., Jin J.-C., Gong L.-K., Lin Y.-P., Du K.-Z., Huang X.-Y. (2021). Co-luminescence in a zero-dimensional organic–inorganic hybrid antimony halide with multiple coordination units. Dalton Trans..

[6] Zhao J.Q., Wang D.Y., Yan T.Y., Wu Y.F., Gong Z.L., Chen Z.W., Yue C.Y., Yan D.P., Lei X.W. (2024). Synchronously Improved Multiple Afterglow and Phosphorescence Efficiencies in 0D Hybrid Zinc Halides With Ultrahigh Anti-Water Stabilities. Angew. Chem. Int. Ed..

[7] Zhuang T., Lin Y., Jin J., Deng Z., Peng Y., Gong L., Wang Z., Du K., Huang X. (2023). A Mechanochemically Synthesized Hybrid Bismuth Halide as Highly Efficient Red Phosphor for Blue Chip-Based WLED. Adv. Opt. Mater..

[8] Zhuang T.-H., Lin Y.-M., Lin H.-W., Guo Y.-L., Li Z.-W., Du K.-Z., Wang Z.-P., Huang X.-Y. (2023). Luminescence Enhancement and Temperature Sensing Properties of Hybrid Bismuth Halides Achieved via Tuning Organic Cations. Molecules.

[9] Quan M.Z., Xiong Y., Xu Y.L., Li M.Y., Chen M.Y., Cao J.D., Zhao J., Liu Q.L. (2024). Structural diversity and photoluminescence enhancement of indium-based hybrid metal halides. J. Alloys Compd..

[10] Wang Z.P., Huang X.Y. (2022). Luminescent Organic-Inorganic Hybrid Metal Halides: An Emerging Class of Stimuli-Responsive Materials. Chem. Eur. J..

[11] Zhou Y.Y., Padture N.P. (2017). Gas-Induced Formation/Transformation of Organic Inorganic Halide Perovskites. ACS Energy Lett..

[12] Liu Y., Huang D.-D., Zhang Z.-Z., Lin H.-W., Du K.-Z., Wang Z.-P., Huang X.-Y. (2024). Modulating organic functional groups in stimuli-responsive luminescent antimony chlorides. J. Mater. Chem. C.

[13] Zhang Z., Lin Y., Jin J., Gong L., Peng Y., Song Y., Shen N., Wang Z., Du K., Huang X. (2021). Crystalline-Phase-Recognition-Induced Domino Phase Transition and Luminescence Switching for Advanced Information Encryption. Angew. Chem. Int. Ed..

[14] Zhang J., Ren M.P., Xu M., Zhang Z.H., An M.X., Lu Y., Lei X.W., Gong Z.L., Yue C.Y. (2024). Ultrafast Visual Detection of a Trace Amount of Water by Highly Efficient Hybrid Manganese Halides. ACS Appl. Mater. Interfaces.

[15] Wang Z.P., Zhang Z.Z., Tao L.Q., Shen N.N., Hu B., Gong L.K., Li J.R., Chen X.P., Huang X.Y. (2019). Hybrid Chloroantimonates(III): Thermally Induced Triple-Mode Reversible Luminescent Switching and Laser- Printable Rewritable Luminescent Paper. Angew. Chem. Int. Ed..

[16] Li D.Y., Wu J.H., Wang X.Y., Zhang X.Y., Yue C.Y., Lei X.W. (2023). Reversible Triple-Mode Photo- and Radioluminescence and Nonlinear Optical Switching in Highly Efficient 0D Hybrid Cuprous Halides. Chem. Mater..

[17] Liu Y.H., Yan X., Xiao L., Jiang W., Liu Q., Liu T.C., Yan T.Y., Yue C.Y., Lei X.W. (2023). Water-Stable 0D Hybrid Manganese Halides with Adjustable Crystal Structure and Emission Color. Adv. Opt. Mater..

[18] Li D.Y., Song J.H., Cheng Y., Wu X.M., Wang Y.Y., Sun C.J., Yue C.Y., Lei X.W. (2022). Ultra-Sensitive, Selective and Repeatable Fluorescence Sensor for Methanol Based on a Highly Emissive 0D Hybrid Lead-Free Perovskite. Angew. Chem. Int. Ed..

[19] Lu X.Y., Peng H., Wei Q.L., Lin W.C., Tian Y., Li T.Z., Zhou S.C., Zhao J.L., Zou B.S. (2023). Bulk assemblies of organic antimony chloride with multiple reversible photoluminescence switching for anti-counterfeiting and information encryption. Mater. Today Phys..

[20] Sun C., Zhang H., Deng Z.H., Fan C., Liu X.H., Luo M.M., Zhao Y.W., Lian K. (2023). Metal-Ion-Doped Manganese Halide Hybrids with Tunable Emission for Advanced Anti-Counterfeiting. Nanomaterials.

[21] Ma W., Qian Q.K., Qaid S.M.H., Zhao S.Y., Liang D.H., Cai W.S., Zang Z.G. (2023). Water-Molecule-Induced Reversible Fluorescence in a One-Dimensional Mn-Based Hybrid Halide for Anticounterfeiting and Digital Encryption-Decryption. Nano Lett..

[22] Jiang R., Peng G.Q., Li Q.J., Wang H.X., Ci Z.P., Wang Q. (2024). Manganese (II) Halides for X-Ray Imaging and Moisture Detection. Adv. Mater. Technol..

[23] Song Z.X., Chen D.P., Yu B.Y., Liu G.K., Li H.Y., Wei Y.Y., Wang S.H., Meng L.Q., Dang Y.Y. (2023). Thermal/Water-Induced Phase Transformation and Photoluminescence of Hybrid Manganese(II)-Based Chloride Single Crystals. Inorg. Chem..

[24] Chen S.Q., Huang M.L., Yin Y.L., Shi J.L. (2023). Paper-based sensor based on lead-free manganese halide for the determination of water content in organic solvents. Microchim. Acta.

[25] Xu W., Li F.M., Cai Z.X., Wang Y.R., Luo F., Chen X. (2016). An ultrasensitive and reversible fluorescence sensor of humidity using perovskite CH_3_NH_3_PbBr_3_. J. Mater. Chem. C.

[26] Qiu Y.X., Ma Z.M., Li Z.W., Sun H.Y., Dai G.K., Fu X.H., Jiang H., Ma Z.Y. (2022). Solely 3-Coordinated Organic-Inorganic Hybrid Copper(I) Halide: Hexagonal Channel Structure, Turn-On Response to Mechanical Force, Moisture, and Amine. Inorg. Chem..

[27] Gao W.R., Leng M.Y., Hu Z.X., Li J.Z., Li D.H., Liu H., Gao L., Niu G.D., Tang J. (2020). Reversible luminescent humidity chromism of organic-inorganic hybrid PEA_2_MnBr_4_ single crystals. Dalton Trans..

[28] Cai W.D., Qin J.J., Pang T.Q., Cai X.Y., Jia R.X., Gao F. (2022). Chirality Induced Crystal Structural Difference in Metal Halide Composites. Adv. Opt. Mater..

[29] Li D.Y., Song J.H., Xu Z.Y., Gao Y.J., Yin X., Hou Y.H., Feng L.J., Yue C.Y., Fei H.H., Lei X.W. (2022). Reversible Triple-Mode Switching in Photoluminescence from 0D Hybrid Antimony Halides. Chem. Mater..

[30] Liu H.L., Ru H.Y., Sun M.E., Wang Z.Y., Zang S.Q. (2022). Organic-Inorganic Manganese Bromide Hybrids with Water-Triggered Luminescence for Rewritable Paper. Adv. Opt. Mater..

[31] Sharma S.K., Phadnis C., Das T.K., Kumar A., Kayaipatti B., Chowdhury A., Yella A. (2019). Reversible Dimensionality Tuning of Hybrid Perovskites with Humidity: Visualization and Application to Stable Solar Cells. Chem. Mater..

[32] Wang Z.P., Huang D.D., Liu Y., Lin H.W., Zhang Z.Z., Ablez A., Zhuang T.H., Du K.Z., Li J., Huang X.Y. (2024). Vacancy Effect on the Luminescent and Water Responsive Properties of Vacancy-Ordered Double Perovskite Derivatives. Angew. Chem. Int. Ed..

[33] Wu J.J., Qi J.L., Guo Y., Yan S.F., Liu W.L., Guo S.P. (2023). Reversible tri-state structural transitions of hybrid copper(i) bromides toward tunable multiple emissions. Inorg. Chem. Front..

[34] Wang Z.P., Xie D.L., Zhang F., Yu J.B., Chen X.P., Wong C.P. (2020). Controlling information duration on rewritable luminescent paper based on hybrid antimony (III) chloride/small-molecule absorbates. Sci. Adv..

[35] Zhao J.Q., Han M.F., Zhao X.J., Ma Y.Y., Jing C.Q., Pan H.M., Li D.Y., Yue C.Y., Lei X.W. (2021). Structural Dimensionality Modulation toward Enhanced Photoluminescence Efficiencies of Hybrid Lead-Free Antimony Halides. Adv. Opt. Mater..

[36] Sun C., Deng Z.Y., Li Z.Y., Chen Z.W., Zhang X.Y., Chen J., Lu H.P., Canepa P., Chen R., Mao L.L. (2023). Achieving Near-unity Photoluminescence Quantum Yields in Organic-Inorganic Hybrid Antimony (III) Chlorides with the SbCl_5_ Geometry. Angew. Chem. Int. Ed..

[37] Liao J.F., Zhang Z.P., Zhou L., Tang Z.K., Xing G.C. (2024). Achieving Near-Unity Red Light Photoluminescence in Antimony Halide Crystals via Polyhedron Regulation. Angew. Chem. Int. Ed..

[38] Lei Y.S., Li Y.H., Lu C.C.F., Yan Q.Z., Wu Y.L., Babbe F., Gong H.X., Zhang S., Zhou J.Y., Wang R.T. (2022). Perovskite superlattices with efficient carrier dynamics. Nature.

[39] Lin J., Zhang Q., Wang L., Liu X.C., Yan W.B., Wu T., Bu X.H., Feng P.Y. (2014). Atomically Precise Doping of Monomanganese Ion into Coreless Supertetrahedral Chalcogenide Nanocluster Inducing Unusual Red Shift in Mn^2+^ Emission. J. Am. Chem. Soc..

[40] Lin F., Wang H., Liu W., Li J. (2020). Zero-dimensional ionic antimony halide inorganic-organic hybrid with strong greenish yellow emission. J. Mater. Chem. C.

[41] Chen D., Dai F.L., Hao S.Q., Zhou G.J., Liu Q.L., Wolverton C., Zhao J., Xia Z.G. (2020). Crystal structure and luminescence properties of lead-free metal halides (C_6_H_5_CH_2_NH_3_)_3_MBr_6_(M = Bi and Sb). J. Mater. Chem. C.

[42] McCall K.M., Stoumpos C.C., Kostina S.S., Kanatzidis M.G., Wessels B.W. (2017). Strong Electron-Phonon Coupling and Self-Trapped Excitons in the Defect Halide Perovskites *A*_3_*M*_2_I_9_ (A = Cs, Rb; M = Bi, Sb). Chem. Mater..

[43] Li Z.Y., Li Y., Liang P., Zhou T.L., Wang L., Xie R.J. (2019). Dual-Band Luminescent Lead-Free Antimony Chloride Halides with Near-Unity Photoluminescence Quantum Efficiency. Chem. Mater..

[44] Feng L.J., Liu H.R., Wang L.L., Yang C.C., Ding Y.W., Lei X.W., Chen Z.W. (2024). Blue light emissive zero-dimensional hybrid cadmium bromide as fluorescence sensor toward benzaldehyde. J. Lumin..

[45] Wang X.C., Bai T.X., Sun J.L., Liu J.Y., Su Y., Chen J.S. (2024). The effect of solvent on the formation of low-dimensional metal halides and their self-trapped exciton emission. Chem. Eng. J..

[46] Xie P.R., Wang P., Zhou J.Q., Guo Z., Mao L.L. (2024). Increasing the Structural Rigidity and Quantum Yield of Highly Emissive Hybrid Antimony Chlorides Using a Diverse Set of Solvent Guests. Adv. Opt. Mater..

[47] Lin J.W., Zhang M.W., Sun N., He S.H., Zhang X.S., Guo Z.N., Zhao J., Liu Q.L., Yuan W.X. (2022). Narrowing the band of green emission in manganese hybrids by reducing the hydrogen bond strength and structural distortion. J. Mater. Chem. C.

[48] Zhang Q., Huang T.W., Liu Z.Y., Feng Y.N., Yu Y., Li L.Y. (2025). Hydrogen bonding evolution and efficient blue light emission in a series of Zn-based organic-inorganic hybrid metal halide crystals. Sci. China Mater..

[49] Huang Z.H., Wang Y.X., Du P., Gao W., Niu P., Xu D.M., Wang L.M., Deng Y.C., Song A.X. (2024). Structural Design of Hybrid Manganese(II) Halides for High Quantum Efficiency and Specific Response to Methanol. Inorg. Chem..

[50] Choi M.H., Moon T.H., Kuk Y., Ok K.M. (2023). Green and Red Photoluminescent Manganese Bromides with Aminomethylpyridine Isomers. Inorg. Chem..

[51] Hu W.H., Xu W.J., Meng Q.R., Zhang X.W., He C.T., Zhang W.X., Chen X.M. (2019). Switching hydrogen bonds to readily interconvert two room-temperature long-term stable crystalline polymorphs in chiral molecular perovskites. Chem. Commun..

[52] Ye H., Hu W.H., Chen X.X., Zhao B.Q., Zhang W.X., Chen X.M. (2023). Heat- and Pressure-driven Room-temperature Polymorphic Transition Accompanied with Switchable SHG Signal in a New Chiral Hexagonal Perovskite. Chem. Asian J..

[53] Sun Z.H., Wang X.Q., Luo J.H., Zhang S.Q., Yuan D.Q., Hong M.C. (2013). Ferroelastic phase transition and switchable dielectric behavior associated with ordering of molecular motion in a perovskite-like architectured supramolecular cocrystal. J. Mater. Chem. C.

[54] Gong Z.L., Zheng W., Huang P., Cheng X.W., Zhang W., Zhang M.R., Han S.Y., Chen X.Y. (2022). Highly efficient Sb^3+^ emitters in 0D cesium indium chloride nanocrystals with switchable photoluminescence through water-triggered structural transformation. Nano Today.

[55] Song G.M., Li M.Z., Zhang S.Z., Wang N.Z., Gong P.F., Xia Z.G., Lin Z.S. (2020). Enhancing Photoluminescence Quantum Yield in 0D Metal Halides by Introducing Water Molecules. Adv. Funct. Mater..

[56] Zhou L., Liao J.F., Huang Z.G., Wei J.H., Wang X.D., Li W.G., Chen H.Y., Kuang D.B., Su C.Y. (2019). A Highly Red-Emissive Lead-Free Indium-Based Perovskite Single Crystal for Sensitive Water Detection. Angew. Chem. Int. Ed..

[57] Luo J.B., Wei J.H., Zhang Z.Z., Kuang D.B. (2022). Water-Molecule-Induced Emission Transformation of Zero-Dimension Antimony-Based Metal Halide. Inorg. Chem..

[58] Sheldrick G.M. (2015). Crystal structure refinement with SHELXL. Acta Crystallogr. C-Struct. Chem..

[59] Spackman M.A., McKinnon J.J. (2002). Fingerprinting intermolecular interactions in molecular crystals. CrystEngComm.

[60] McKinnon J.J., Spackman M.A., Mitchell A.S. (2004). Novel tools for visualizing and exploring intermolecular interactions in molecular crystals. Acta. Crystallogr. B. Struct. Sci. Cryst. Eng. Mater..

[61] McKinnon J.J., Jayatilaka D., Spackman M.A. (2007). Towards quantitative analysis of intermolecular interactions with Hirshfeld surfaces. Chem. Commun..

[62] Spackman M.A., McKinnon J.J., Jayatilaka D. (2008). Electrostatic potentials mapped on Hirshfeld surfaces provide direct insight into intermolecular interactions in crystals. CrystEngComm.

[63] Spackman M. (2014). Rationalising molecular crystal structures using Hirshfeld surfaces. Acta Cryst..

[64] Turner M.J., McKinnon J.J., Jayatilaka D., Spackman M.A. (2011). Visualisation and characterisation of voids in crystalline materials. CrystEngComm.

