# Supplementary material for: Humidity-Triggered Reversible 0–1D Phase Transition in Hybrid Antimony Halides
Source: Nanomaterials (Basel). 2025 Mar 14;15(6):442. doi: 10.3390/nano15060442 (PMC11945002; doi:10.3390/nano15060442)

checkCIF/PLATON report

Structure factors have been supplied for datablock(s) x

THIS REPORT IS FOR GUIDANCE ONLY. IF USED AS PART OF A REVIEW PROCEDURE FOR PUBLICATION, IT SHOULD NOT REPLACE THE EXPERTISE OF AN EXPERIENCED CRYSTALLOGRAPHIC REFEREE.

No syntax errors found. CIF dictionary Interpreting this report

Datablock: x

Bond precision:	C-C = 0.0064 A		Wavelength=0.71073
Cell:	a=8.6622(3)	b=8.8904(3)	c=33.1475(11)
	alpha=90	beta=90	gamma=90
Temperature:	100 K		

	Calculated	Reported
Volume	2552.70(15)	2552.70(15)
Space group	P 21 21 21	P 21 21 21
Hall group	P 2ac 2ab	P 2ac 2ab
Moiety formula	Cl6 Sb, 3(C4 H10 N O), C2 H3 N	?
Sum formula	C14 H33 Cl6 N4 O3 Sb	C14 H33 Cl6 N4 O3 Sb
Mr	639.90	639.89
Dx,g cm-3	1.665	1.665
Z	4	4
Mu (mm-1)	1.731	1.731
F000	1288.0	1288.0
F000’	1289.52	
h,k,lmax	12,12,47	11,12,42
Nref	8023[4531]	6066
Tmin,Tmax	0.655,0.707	0.730,1.000
Tmin’	0.642	

Correction method= # Reported T Limits: Tmin=0.730 Tmax=1.000
AbsCorr = NONE

Data completeness= 1.34/0.76 Theta(max)= 30.830

R(reflections)= 0.0294(5436) wR2(reflections)= 0.0604(6066)

S = 1.060 Npar= 273

The following ALERTS were generated. Each ALERT has the format
test-name_ALERT_alert-type_alert-level.
Click on the hyperlinks for more details of the test.

Alert level C

```
PLAT915_ALERT_3_C No Flack x Check Done: Low Friedel Pair Coverage         68 %
```

Alert level G

```
PLAT398_ALERT_2_G Deviating  C-O-C   Angle From 120 for O2       .      109.1 Degree
PLAT398_ALERT_2_G Deviating  C-O-C   Angle From 120 for O3       .      109.3 Degree
PLAT480_ALERT_4_G Long H...A H-Bond Reported H1B      ..CL3      .       2.94 Ang.
PLAT480_ALERT_4_G Long H...A H-Bond Reported H3A      ..CL2      .       2.93 Ang.
PLAT480_ALERT_4_G Long H...A H-Bond Reported H3A      ..CL6      .       2.97 Ang.
PLAT480_ALERT_4_G Long H...A H-Bond Reported H4A      ..CL2      .       2.93 Ang.
PLAT480_ALERT_4_G Long H...A H-Bond Reported H4B      ..CL4      .       2.84 Ang.
PLAT480_ALERT_4_G Long H...A H-Bond Reported H5B      ..CL4      .       2.99 Ang.
PLAT480_ALERT_4_G Long H...A H-Bond Reported H6B      ..O3       .       2.62 Ang.
PLAT480_ALERT_4_G Long H...A H-Bond Reported H8B      ..CL1      .       2.90 Ang.
PLAT480_ALERT_4_G Long H...A H-Bond Reported H10A     ..CL1      .       2.88 Ang.
PLAT480_ALERT_4_G Long H...A H-Bond Reported H10A     ..CL5      .       2.92 Ang.
PLAT480_ALERT_4_G Long H...A H-Bond Reported H12A     ..N4       .       2.70 Ang.
PLAT480_ALERT_4_G Long H...A H-Bond Reported H14B     ..CL2      .       2.92 Ang.
PLAT794_ALERT_5_G Tentative Bond Valency for Sb1       (III)     .       2.77 Info
PLAT883_ALERT_1_G Absent Datum for _atom_sites_solution_primary ..     Please Do !
PLAT899_ALERT_4_G SHELXL2018 is Outdated   and Succeeded by SHELXL     2019/3 Note
PLAT910_ALERT_3_G Missing # of FCF Reflection(s) Below Theta(Min).          1 Note
                0  0  2,
PLAT912_ALERT_4_G Missing # of FCF Reflections Above STh/L=  0.600        705 Note
PLAT933_ALERT_2_G Number of HKL-OMIT Records in Embedded .res File          2 Note
               -7  3 19,   9  1 10,
PLAT941_ALERT_3_G Average HKL Measurement Multiplicity ..........         4.6 Low
PLAT952_ALERT_5_G Calculated (ThMax) and CIF-Reported Lmax Differ.          5 Units
PLAT958_ALERT_1_G Calculated (ThMax) and Actual (FCF) Lmax Differ.          5 Units
PLAT969_ALERT_5_G The 'Henn et al.' R-Factor-gap value ..........       1.371 Note
              Predicted wR2: Based on SigI**2  4.40 or SHELX Weight  5.69
PLAT978_ALERT_2_G Number C-C Bonds with Positive Residual Density.          0 Info
```

0 **ALERT level A** = Most likely a serious problem - resolve or explain
0 **ALERT level B** = A potentially serious problem, consider carefully
1 **ALERT level C** = Check. Ensure it is not caused by an omission or oversight
25 **ALERT level G** = General information/check it is not something unexpected

2 ALERT type 1 CIF construction/syntax error, inconsistent or missing data
4 ALERT type 2 Indicator that the structure model may be wrong or deficient
3 ALERT type 3 Indicator that the structure quality may be low
14 ALERT type 4 Improvement, methodology, query or suggestion
3 ALERT type 5 Informative message, check

It is advisable to attempt to resolve as many as possible of the alerts in all categories. Often the minor alerts point to easily fixed oversights, errors and omissions in your CIF or refinement strategy, so attention to these fine details can be worthwhile. In order to resolve some of the more serious problems it may be necessary to carry out additional measurements or structure refinements. However, the purpose of your study may justify the reported deviations and the more serious of these should normally be commented upon in the discussion or experimental section of a paper or in the "special_details" fields of the CIF. checkCIF was carefully designed to identify outliers and unusual parameters, but every test has its limitations and alerts that are not important in a particular case may appear. Conversely, the absence of alerts does not guarantee there are no aspects of the results needing attention. It is up to the individual to critically assess their own results and, if necessary, seek expert advice.

Publication of your CIF in IUCr journals

A basic structural check has been run on your CIF. These basic checks will be run on all CIFs submitted for publication in IUCr journals (*Acta Crystallographica*, *Journal of Applied Crystallography*, *Journal of Synchrotron Radiation*); however, if you intend to submit to *Acta Crystallographica Section C* or *E* or *IUCrData*, you should make sure that full publication checks are run on the final version of your CIF prior to submission.

Publication of your CIF in other journals

Please refer to the *Notes for Authors* of the relevant journal for any special instructions relating to CIF submission.

PLATON version of 02/02/2025; check.def file version of 02/02/2025

Datablock x - ellipsoid plot

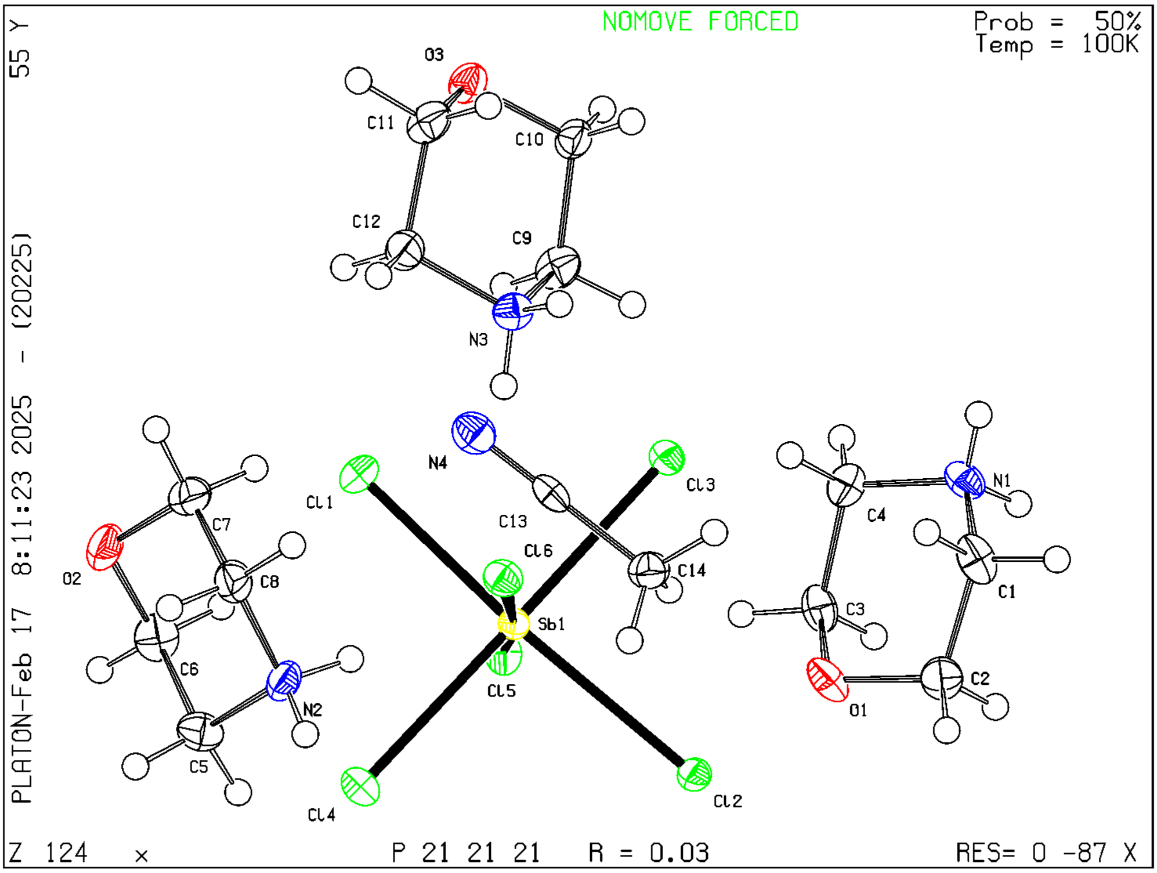

Supplement: Supplementary file 1 [file nanomaterials-15-00442-s001.zip › checkcif-YI3-34A.pdf]
