# Supplementary material for: Humidity-Triggered Reversible 0–1D Phase Transition in Hybrid Antimony Halides
Source: Nanomaterials (Basel). 2025 Mar 14;15(6):442. doi: 10.3390/nano15060442 (PMC11945002; doi:10.3390/nano15060442)

checkCIF/PLATON report

Structure factors have been supplied for datablock(s) x

THIS REPORT IS FOR GUIDANCE ONLY. IF USED AS PART OF A REVIEW PROCEDURE FOR PUBLICATION, IT SHOULD NOT REPLACE THE EXPERTISE OF AN EXPERIENCED CRYSTALLOGRAPHIC REFEREE.

No syntax errors found. CIF dictionary Interpreting this report

Datablock: x

Bond precision: C-C = 0.0065 A Wavelength=0.71073

Cell:	a=9.0378(4)	b=10.1349(4)	c=17.8262(6)
	alpha=90	beta=90	gamma=90
Temperature:	100 K		

	Calculated	Reported
Volume	1632.83(11)	1632.83(11)
Space group	P 21 21 21	P 21 21 21
Hall group	P 2ac 2ab	P 2ac 2ab
Moiety formula	Cl5 Sb, 2(C4 H10 N O)	?
Sum formula	C8 H20 Cl5 N2 O2 Sb	C8 H20 Cl5 N2 O2 Sb
Mr	475.27	475.26
Dx,g cm-3	1.933	1.933
Z	4	4
Mu (mm-1)	2.503	2.503
F000	936.0	936.0
F000’	936.83	
h,k,lmax	12,14,25	12,13,24
Nref	5017[2844]	3907
Tmin,Tmax	0.582,0.591	0.631,1.000
Tmin’	0.571	

Correction method= # Reported T Limits: Tmin=0.631 Tmax=1.000
AbsCorr = NONE

Data completeness= 1.37/0.78 Theta(max)= 30.562

R(reflections)= 0.0274(3613) wR2(reflections)= 0.0572(3907)

S = 1.026 Npar= 176

The following ALERTS were generated. Each ALERT has the format
test-name_ALERT_alert-type_alert-level.
Click on the hyperlinks for more details of the test.

Alert level C

```
PLAT915_ALERT_3_C No Flack x Check Done: Low Friedel Pair Coverage         70 %
```

Alert level G

```
PLAT002_ALERT_2_G Number of Distance or Angle Restraints on AtSite          2 Note
PLAT172_ALERT_4_G The CIF-Embedded .res File Contains DFIX Records          1 Report
PLAT480_ALERT_4_G Long H...A H-Bond Reported H1C      ..CL1      .       2.99 Ang.
PLAT480_ALERT_4_G Long H...A H-Bond Reported H1D      ..CL1      .       2.95 Ang.
PLAT480_ALERT_4_G Long H...A H-Bond Reported H1D      ..CL3      .       2.96 Ang.
PLAT480_ALERT_4_G Long H...A H-Bond Reported H2C      ..CL3      .       2.93 Ang.
PLAT480_ALERT_4_G Long H...A H-Bond Reported H3B      ..CL2      .       2.97 Ang.
PLAT480_ALERT_4_G Long H...A H-Bond Reported H4A      ..CL2      .       2.93 Ang.
PLAT480_ALERT_4_G Long H...A H-Bond Reported H5A      ..CL2      .       2.88 Ang.
PLAT480_ALERT_4_G Long H...A H-Bond Reported H5A      ..CL4      .       2.84 Ang.
PLAT480_ALERT_4_G Long H...A H-Bond Reported H6A      ..CL4      .       2.92 Ang.
PLAT480_ALERT_4_G Long H...A H-Bond Reported H6B      ..CL2      .       2.99 Ang.
PLAT480_ALERT_4_G Long H...A H-Bond Reported H6B      ..CL5      .       2.89 Ang.
PLAT480_ALERT_4_G Long H...A H-Bond Reported H7A      ..CL1      .       2.95 Ang.
PLAT480_ALERT_4_G Long H...A H-Bond Reported H7A      ..CL3      .       2.92 Ang.
PLAT480_ALERT_4_G Long H...A H-Bond Reported H8A      ..CL5      .       2.90 Ang.
PLAT794_ALERT_5_G Tentative Bond Valency for Sb1       (III)     .       2.77 Info
PLAT860_ALERT_3_G Number of Least-Squares Restraints ............           1 Note
PLAT883_ALERT_1_G Absent Datum for _atom_sites_solution_primary ..     Please Do !
PLAT899_ALERT_4_G SHELXL2018 is Outdated   and Succeeded by SHELXL     2019/3 Note
PLAT912_ALERT_4_G Missing # of FCF Reflections Above STh/L=  0.600        418 Note
PLAT941_ALERT_3_G Average HKL Measurement Multiplicity ..........         4.6 Low
PLAT969_ALERT_5_G The 'Henn et al.' R-Factor-gap value ..........       1.427 Note
              Predicted wR2: Based on SigI**2  4.01 or SHELX Weight  5.57
PLAT978_ALERT_2_G Number C-C Bonds with Positive Residual Density.          0 Info
```

```
   0 ALERT level A = Most likely a serious problem - resolve or explain
   0 ALERT level B = A potentially serious problem, consider carefully
   1 ALERT level C = Check. Ensure it is not caused by an omission or oversight
  24 ALERT level G = General information/check it is not something unexpected

   1 ALERT type 1 CIF construction/syntax error, inconsistent or missing data
   2 ALERT type 2 Indicator that the structure model may be wrong or deficient
   3 ALERT type 3 Indicator that the structure quality may be low
  17 ALERT type 4 Improvement, methodology, query or suggestion
   2 ALERT type 5 Informative message, check
```

It is advisable to attempt to resolve as many as possible of the alerts in all categories. Often the minor alerts point to easily fixed oversights, errors and omissions in your CIF or refinement strategy, so attention to these fine details can be worthwhile. In order to resolve some of the more serious problems it may be necessary to carry out additional measurements or structure refinements. However, the purpose of your study may justify the reported deviations and the more serious of these should normally be commented upon in the discussion or experimental section of a paper or in the "special_details" fields of the CIF. checkCIF was carefully designed to identify outliers and unusual parameters, but every test has its limitations and alerts that are not important in a particular case may appear. Conversely, the absence of alerts does not guarantee there are no aspects of the results needing attention. It is up to the individual to critically assess their own results and, if necessary, seek expert advice.

Publication of your CIF in IUCr journals

A basic structural check has been run on your CIF. These basic checks will be run on all CIFs submitted for publication in IUCr journals (*Acta Crystallographica*, *Journal of Applied Crystallography*, *Journal of Synchrotron Radiation*); however, if you intend to submit to *Acta Crystallographica Section C* or *E* or *IUCrData*, you should make sure that full publication checks are run on the final version of your CIF prior to submission.

Publication of your CIF in other journals

Please refer to the *Notes for Authors* of the relevant journal for any special instructions relating to CIF submission.

PLATON version of 02/02/2025; check.def file version of 02/02/2025

Datablock x - ellipsoid plot

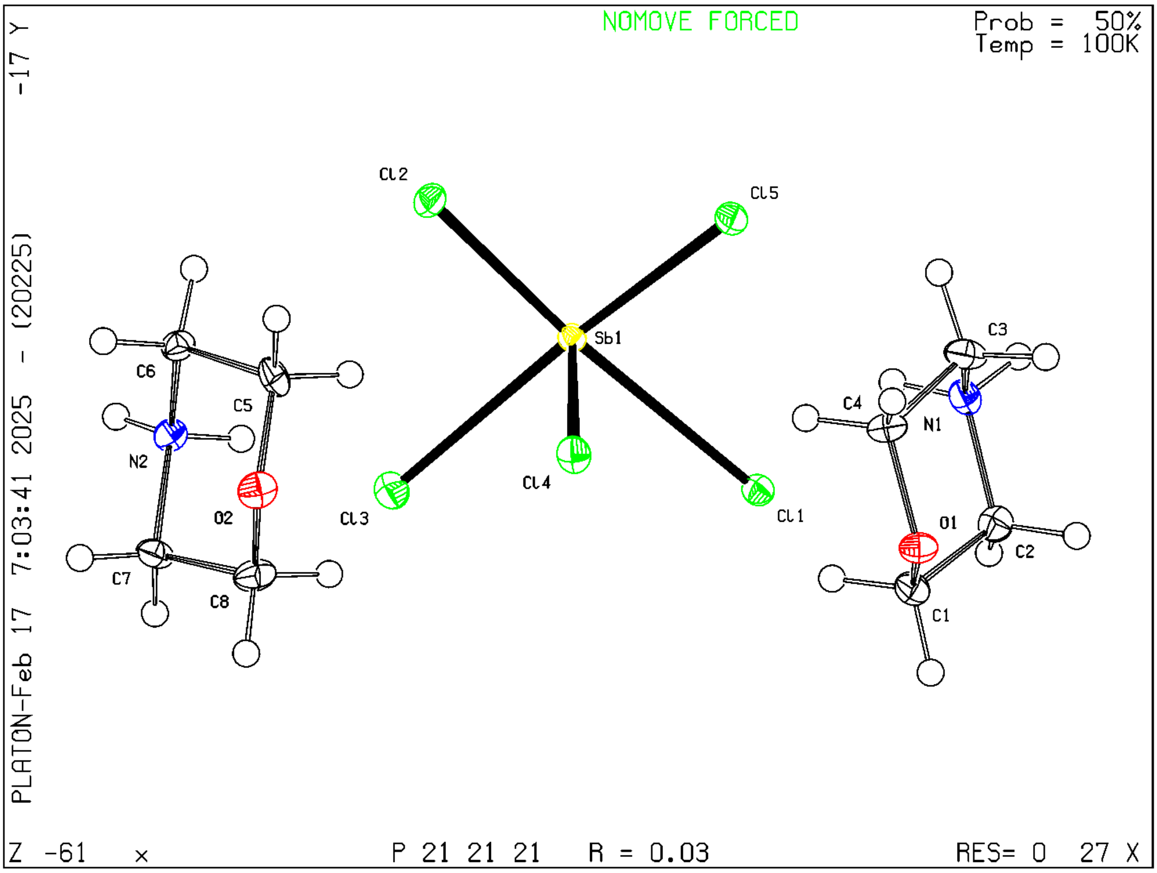

Supplement: Supplementary file 1 [file nanomaterials-15-00442-s001.zip › checkcif-YI3-37C.pdf]
