# Supplementary material for: Humidity-Triggered Reversible 0–1D Phase Transition in Hybrid Antimony Halides
Source: Nanomaterials (Basel). 2025 Mar 14;15(6):442. doi: 10.3390/nano15060442 (PMC11945002; doi:10.3390/nano15060442)
Supplement: Supplementary file 1 [file nanomaterials-15-00442-s001.zip › SI-Revised.pdf]

Humidity-triggered reversible phase transition fluorescence response of 0D-1D antimony-based hybrid halides

Yi Liu [1,2,3], Jia-Hua Luo [1,2,3], Abdusalam Ablez [1,2,3], Jin-Mei Liu [1,2,3], Nian-Hao Wang [1,2,3], Hao-Wei Lin [2], Ze-Ping Wang [2,*] and Xiao-Ying Huang [2,*]

1 College of Chemistry, Fuzhou University, Fuzhou 350108 (P. R. China); yiliu@fjirsm.ac.cn, luojiahua@fjirsm.ac.cn, liujinmei@fjirsm.ac.cn, wangnianhao@fjirsm.ac.cn, abdslm@fjirsm.ac.cn.

2 State Key Laboratory of Structural Chemistry, Fujian Institute of Research on the Structure of Matter, Chinese Academy of Sciences, Fuzhou, Fujian 350002, P. R. China; yiliu@fjirsm.ac.cn, luojiahua@fjirsm.ac.cn, liujinmei@fjirsm.ac.cn, wangnianhao@fjirsm.ac.cn, abdslm@fjirsm.ac.cn, linhw@fjirsm.ac.cn, wzping520@msn.cn, xyhuang@fjirsm.ac.cn.

3 Fujian College, University of Chinese Academy of Sciences, Fuzhou 350002, P. R. China; xyhuang@fjirsm.ac.cn.

* Correspondence: xyhuang@fjirsm.ac.cn (X.-Y.H.); wzping520@msn.cn (Z.-P. W.); Tel.: +86-591-6317-3145 (X.-17 Y.H.)

Table S1. Crystal data and structure refinement details for $(Mp)_3SbCl_6{\cdot}MeCN$ and $(Mp)_2SbCl_5$.

	$(Mp)_3SbCl_6{\cdot}MeCN$	$(Mp)_2SbCl_5$
CCDC number	2424336	2424337
Empirical formula	$C_{14}H_{33}Cl_6N_4O_3Sb$	$C_8H_{20}N_2O_2Cl_5Sb$
Formula weight	639.90	475.26
Temperature/K	100(2)	100(2)
Wavelength/Å	0.71073	0.71073
Crystal system	orthorhombic	orthorhombic
Space group	$P2_12_12_1$	$P2_12_12_1$
Flack	-0.04(2)	-0.02(2)
a/Å	8.6622(3)	9.0378(4)
b/Å	8.8904(3)	10.1349(4)
c/Å	33.1475(11)	17.8262(6)
α/°	90	90
β/°	90	90
γ/°	90	90
Volume/Å^3	2552.7(15)	1632.83(11)
Z	4	4
ρ_{calc} g/cm^3	1.665	1.933
Absorption coefficient/mm^{-1}	1.2731	2.503
F(000)	1288.0	936.0
Crystal size/mm^3	0.25 × 0.21 × 0.20	0.22 × 0.22 × 0.21
Theta range for data collection /°	4.744-61.66	4.57-61.124
Reflections collected/ unique	17121/6066 [R_{int} = 0.0405]	10947/3907 [R_{int} = 0.0376]
Refinement method	Full-matrix least-squares on F^2	Full-matrix least-squares on F^2
Data/restraints/parameters	6066/0/273	3907/1/176
Goodness-of-fit on F^2	1.060	1.026
Final R indexes [I >= 2σ (I)]	R_1 = 0.029, wR_2 = 0.0574	R_1 = 0.0274, wR_2 = 0.0558
Final R indexes [all data]	R_1 = 0.03778, wR_2 = 0.0604	R_1 = 0.0322, wR2 = 0.0572
Largest diff. peak and hole	0.55/-0.65	0.80/-0.76

[a] $R_1 = \sum \| F_o | - | F_c \| / \sum | F_o |$, [b] $wR_2 = [\sum w(F_o^2 - F_c^2)^2 / \sum w(F_o^2)^2]^{1/2}$

Table S2. Selected bond length (Å) and bond angle (°) for $(Mp)_3SbCl_6·MeCN$ and $(Mp)_2SbCl_5$.

**(Mp)**$_3$SbCl$_6$·MeCN			
Sb(1)-Cl(1)	2.5220(10)	Sb(1)-Cl(4)	2.6241(11)
Sb(1)-Cl(2)	2.7463(9)	Sb(1)-Cl(5)	2.4531(11)
Sb(1)-Cl(3)	2.6731(10)	Sb(1)-Cl(6)	3.0518(11)
Cl(1)-Sb(1)-Cl(2)	171.28(4)	Cl(4)-Sb(1)-Cl(3)	175.86(4)
Cl(1)-Sb(1)-Cl(3)	87.87(4)	Cl(4)-Sb(1)-Cl(6)	90.01(3)
Cl(1)-Sb(1)-Cl(4)	91.07(4)	Cl(5)-Sb(1)-Cl(2)	84.03(4)
Cl(1)-Sb(1)-Cl(6)	85.94(3)	Cl(5)-Sb(1)-Cl(3)	88.20(3)
Cl(2)-Sb(1)-Cl(6)	101.30(3)	Cl(5)-Sb(1)-Cl(1)	88.91(4)
Cl(3)-Sb(1)-Cl(2)	86.81(3)	Cl(5)-Sb(1)-Cl(4)	87.78(11)
Cl(3)-Sb(1)-Cl(6)	93.91(3)	Cl(5)-Sb(1)-Cl(6)	174.35(3)
Cl(4)-Sb(1)-Cl(2)	93.75(3)		

Symmetry transformations used to generate equivalent atoms: N.A. for $(Mp)_3SbCl_6·MeCN$.

(Mp)$_2$SbCl$_5$			
Sb(1)-Cl(1)	2.8929(11)	Sb(1)-Cl(3)	2.8424(12)
Sb(1)-Cl(3)#1	3.2866(12)	Sb(1)-Cl(4)	2.4133(12)
Sb(1)-Cl(2)	2.4440(11)	Sb(1)-Cl(5)	2.5071(11)
Cl(1)-Sb(1)-Cl(3)#1	93.16(3)	Cl(4)-Sb(1)-Cl(2)	93.68(4)
Cl(2)-Sb(1)-Cl(1)	173.72(3)	Cl(4)-Sb(1)-Cl(3)	84.02(4)
Cl(2)-Sb(1)-Cl(3)	90.79(4)	Cl(4)-Sb(1)-Cl(3)#1	177.17(4)
Cl(2)-Sb(1)-Cl(3)#1	89.14(3)	Cl(4)-Sb(1)-Cl(5)	87.79(4)
Cl(2)-Sb(1)-Cl(5)	89.34(4)	Cl(5)-Sb(1)-Cl(1)	84.75(3)
Cl(3)-Sb(1)-Cl(1)	94.76(3)	Cl(5)-Sb(1)-Cl(3)	171.80(4)
Cl(3)-Sb(1)-Cl(1)#1	96.147(9)	Cl(5)-Sb(1)-Cl(3)#1	92.05(4)
Cl(4)-Sb(1)-Cl(1)	84.01(4)		

Symmetry transformations used to generate equivalent atoms: N.A. for $(Mp)_2SbCl_5$.

Table S3. Hydrogen bonds data for $(Mp)_3SbCl_6{\cdot}MeCN$ and $(Mp)_2SbCl_5$.

D-H···A	d(D-H)	d(H···A)	d(D···A)	<(DHA)
**$(Mp)_3SbCl_6{\cdot}MeCN$**				
N(1)-H(1C)···Cl(2)#1	0.82(5)	2.38(5)	3.141(4)	155(4)
N(1)-H(1D)···Cl(2)#2	0.96(5)	2.59(5)	3.335(4)	134(4)
N(1)-H(1D)···Cl(3)#2	0.96(5)	2.63(5)	3.338(4)	131(4)
C(1)-H(1A)···O(1)#3	0.99	2.45	3.177(6)	129.6
C(1)-H(1B)···Cl(3)#2	0.99	2.94	3.507(5)	117.5
C(3)-H(3A)···Cl(2)	0.99	2.93	3.530(4)	119.7
C(3)-H(3A)···Cl(6)	0.99	2.97	3.759(5)	137.2
C(4)-H(4A)···Cl(2)#2	0.99	2.93	3.459(5)	114.5
C(4)-H(4B)···Cl(4)#1	0.99	2.84	3.721(4)	148
N(2)-H(2C)···Cl(3)#4	0.87(6)	2.42(5)	3.220(4)	153(4)
N(2)-H(2D)···Cl(6)	0.92(5)	2.23(6)	3.145(5)	171(4)
C(5)-H(5B)···Cl(4)	0.99	2.99	3.724(5)	132.1
C(6)-H(6B)···O(3)#5	0.99	2.62	3.448(6)	141
C(7)-H(7A)···Cl(1)#5	0.99	2.67	3.499(5)	141.7
C(8)-H(8A)···N(4)	0.99	2.57	3.509(6)	158.6
C(8)-H(8B)···Cl(1)#4	0.99	2.9	3.627(5)	131.1
N(3)-H(3C)···Cl(4)#1	0.85(5)	2.44(5)	3.258(4)	162(5)
N(3)-H(3D)···Cl(6)	0.88(5)	2.26(5)	3.139(4)	173(5)
C(10)-H(10A)···Cl(1)#1	0.99	2.88	3.786(5)	153
C(10)-H(10A)···Cl(5)#1	0.99	2.92	3.592(4)	126.1
C(10)-H(10B)···N(4)#6	0.99	2.5	3.463(7)	164.9
C(12)-H(12A)···O(3)#7	0.99	2.59	3.281(6)	126.6
C(12)-H(12A)···N(4)	0.99	2.7	3.403(6)	128.5
C(14)-H(14B)···Cl(2)#3	0.98	2.92	3.526(4)	121

Symmetry transformations used to generate equivalent atoms:
#1 +*x*, 1+*y*, +*z*; #2 -*x*, 1/2+*y*, 1/2-*z*; #3 1-*x*, 1/2+*y*, 1/2-*z*; #4 1+*x*, +*y*, +*z*; #5 1/2+*x*, 1/2-*y*, 1-*z*; #6 -1+*x*, +*y*, +*z*; #7 1/2+*x*, 3/2-*y*, 1-*z*.

D-H···A	d(D-H)	d(H···A)	d(D···A)	<(DHA)
**(Mp)**$_2$SbCl$_5$.				
N(1)-H(1A)···Cl(1)	0.90(6)	2.71(5)	3.394(4)	133(4)
N(1)-H(1A)···Cl(5)	0.90(6)	2.65(5)	3.402(4)	141(4)
N(1)-H(1B)···Cl(1)#1	0.93(6)	2.34(6)	3.175(4)	149(4)
C(1)-H(1C)···Cl(1)	0.99	2.99	3.679(5)	127.7
C(1)-H(1D)···Cl(1)#2	0.99	2.95	3.519(5)	117.8
C(1)-H(1D)···Cl(3)#3	0.99	2.96	3.603(5)	123.4
C(1)-H(1D)···Cl(5)#2	0.99	2.78	3.517(5)	131.8
C(2)-H(2C)···Cl(3)#1	0.99	2.93	3.827(5)	150.6
C(3)-H(3B)···Cl(2)#3	0.99	2.97	3.759(5)	137.2
C(4)-H(4A)···Cl(2)#4	0.99	2.93	3.437(4)	112.5
N(2)-H(2A)···Cl(3)	0.95(5)	2.37(5)	3.294(4)	165(5)
N(2)-H(2B)···Cl(1)#5	0.864(13)	2.73(4)	3.347(4)	129(4)
N(2)-H(2B)···O(1)#6	0.864(13)	2.38(5)	2.811(5)	111(4)
C(5)-H(5A)···Cl(2)	0.99	2.88	3.569(5)	127.5
C(5)-H(5A)···Cl(4)	0.99	2.84	3.766(5)	155.9
C(5)-H(5B)···Cl(1)#7	0.99	2.75	3.638(4)	149.9
C(6)-H(6A)···Cl(4)#8	0.99	2.92	3.799(5)	148.3
C(6)-H(6B)···Cl(2)	0.99	2.99	3.613(5)	122.3
C(6)-H(6B)···Cl(5)#9	0.99	2.89	3.587(5)	128.3
C(7)-H(7A)···Cl(1)#5	0.99	2.95	3.469(5)	113.4
C(7)-H(7A)···Cl(3)#5	0.99	2.92	3.833(5)	154.4
C(8)-H(8A)···Cl(5)#10	0.99	2.9	3.754(5)	144.7

Symmetry transformations used to generate equivalent atoms:
#1 1/2+*x*, 1/2-*y*, -*z*; #2 -1/2+*x*, 1/2-*y*, -*z*; # 3+*x*, -1+*y*, +*z*; #4 1-*x*, -1/2+*y*, 1/2-*z*; #5 -1/2+*x*, 3/2-*y*, -*z*; #6 +*x*, 1+*y*, +*z*; #7 1/2-*x*, 1-*y*, 1/2+*z*; #8 -*x*, 1/2+*y*, 1/2-*z*; #9 1-*x*, 1/2+*y*, 1/2-*z*; #10 -1+*x*, +*y*, +*z*

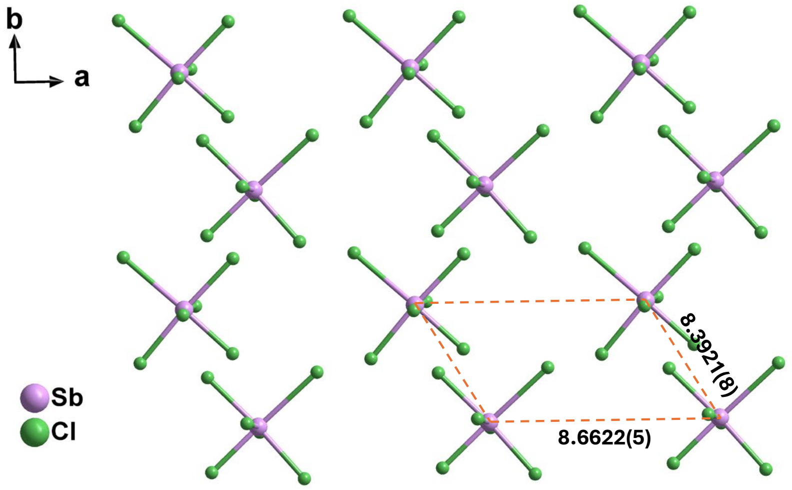

Figure S1. The Sb···Sb distances in the anion part of $(Mp)_3SbCl_6{\cdot}MeCN$.

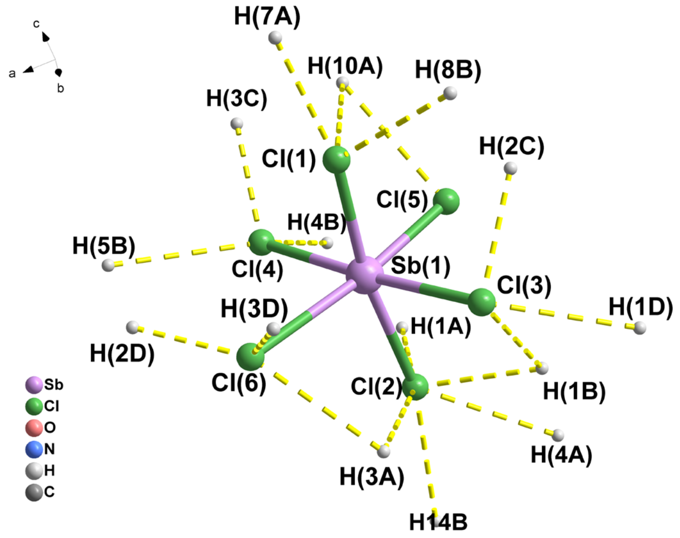

Figure S2 Hydrogen bonds in the $[SbCl_6]^{3-}$ anion of $(Mp)_3SbCl_6{\cdot}MeCN$.

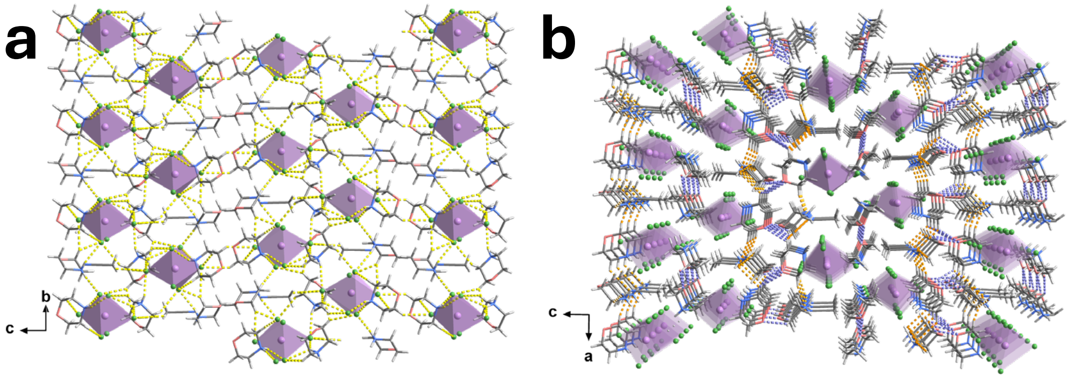

Figure S3. (a) The $(Mp)_3SbCl_6{\cdot}MeCN$ is connected by C-H···Cl and N-H···Cl along the *a*-axis direction to form a two-dimensional layered structure. Yellow dotted lines indicate hydrogen bonds. (b) The three-dimensional supramolecular structure formed by C-H···N (orange dotted line) and C-H···O (blue dotted line) hydrogen bonds along the *b*-axis direction.

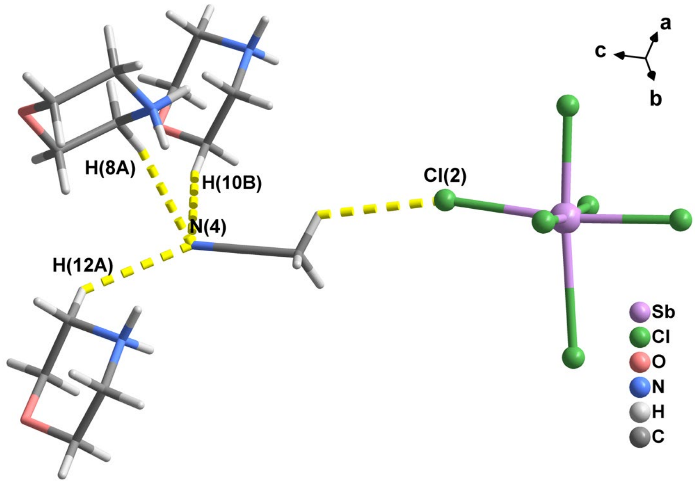

Figure S4. Hydrogen bonds connected with MeCN in $(Mp)_3SbCl_6{\cdot}MeCN$.

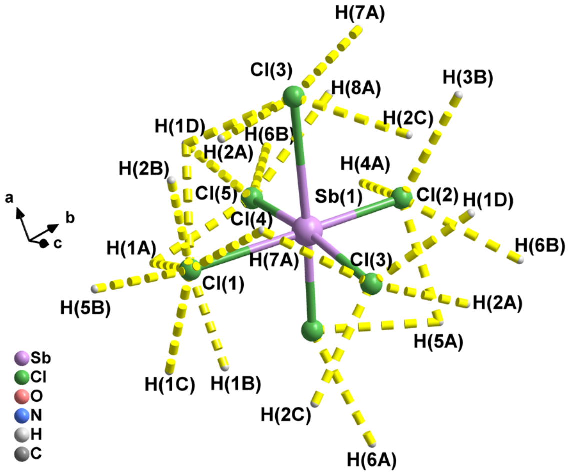

Figure S5. Hydrogen bonds in the $[SbCl_6]^{3-}$ anion of $(Mp)_2SbCl_5$.

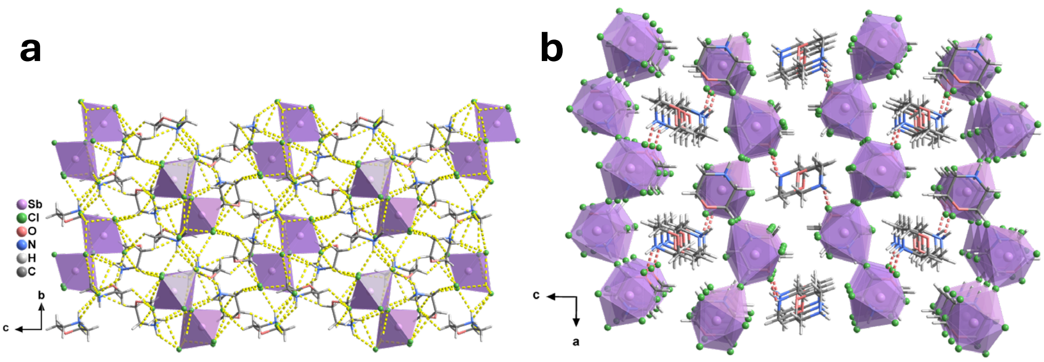

Figure S6. (a) The $(Mp)_2SbCl_5$ is connected by C-H⋯Cl and N-H⋯Cl along the *a*-axis direction to form a two-dimensional layered structure. Yellow dotted lines indicate hydrogen bonds. (b) The three-dimensional supramolecular structure formed by N-H⋯O (pink dotted line) hydrogen bonds along the *b*-axis direction.

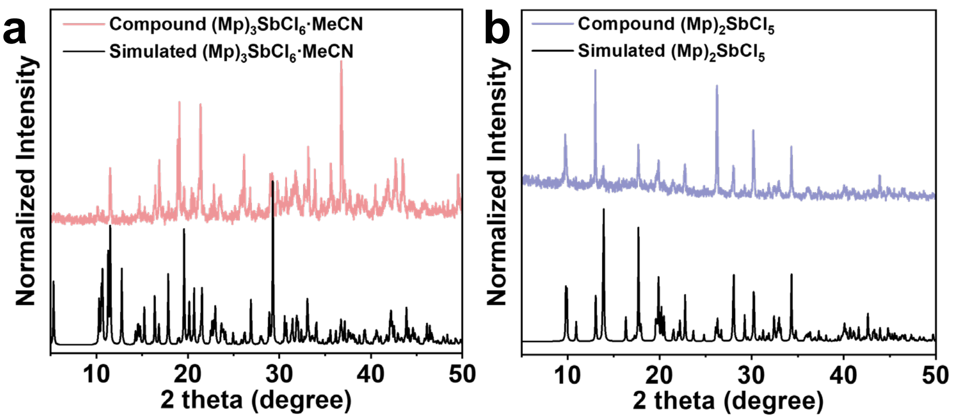

Figure S7. PXRD patterns of $(Mp)_3SbCl_6\cdot MeCN$ (a), and $(Mp)_2SbCl_5$ (b).

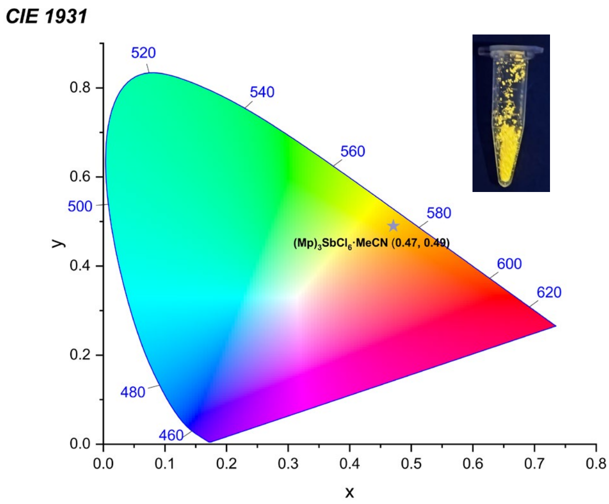

Figure S8. CIE chromaticity coordinates corresponding to $(Mp)_3SbCl_6\cdot MeCN$. The illustration shows $(Mp)_3SbCl_6\cdot MeCN$ emitting orange-yellow fluorescence under a 365 nm ultraviolet lamp.

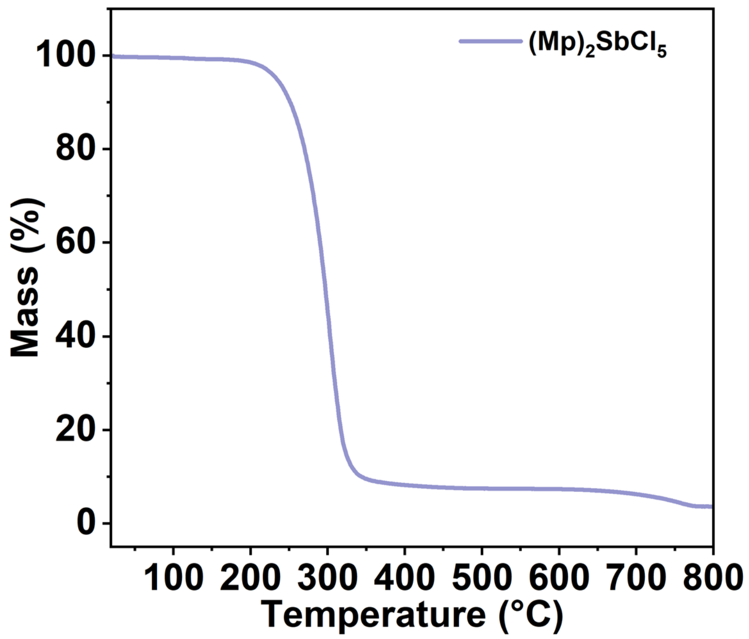

Figure S9. Thermogravimetric (TG) curve of $(Mp)_2SbCl_5$.

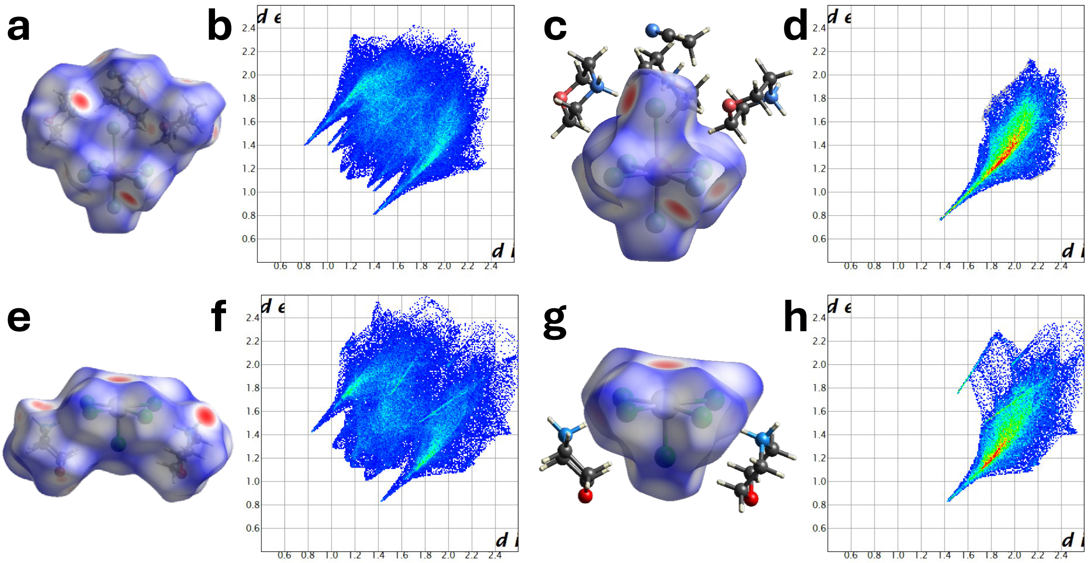

Figure S10. (a) Hirshfeld surface analysis of $(Mp)_3SbCl_6$·MeCN. (b) 2D fingerprint plot of $(Mp)_3SbCl_6$·MeCN. (c) Hirshfeld surface analysis of the $[SbCl_6]^{3-}$ anion part of $(Mp)_3SbCl_6$·MeCN. (d) 2D fingerprint plot of the $[SbCl_6]^{3-}$ anion part of $(Mp)_3SbCl_6$·MeCN. (e) Hirshfeld surface analysis of $(Mp)_2SbCl_5$. (f) 2D fingerprint plot of $(Mp)_2SbCl_5$. (g) Hirshfeld surface analysis of the $[SbCl_5]^{2-}$ anion part of $(Mp)_2SbCl_5$. (h) 2D fingerprint plot of the $[SbCl_5]^{2-}$ anion part of $(Mp)_2SbCl_5$.

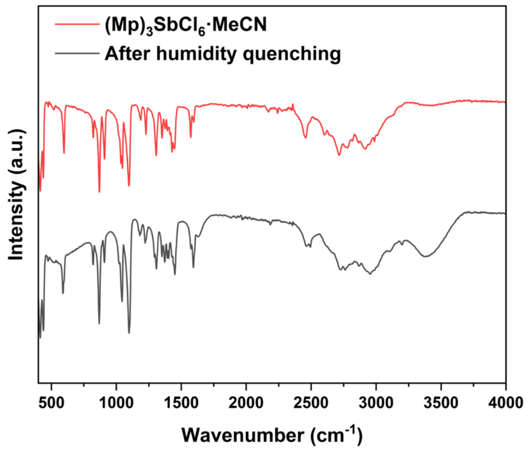

Figure S11. Infrared spectra of (Mp)$_3$SbCl$_6$·MeCN before and after humidity quenching.

We also tested their FTIR. As shown in Figure S11, the curve in the 3225-3725 cm^{-1} region significantly strengthens after humidity quenching, with the appearance of characteristic peaks. This change can be attributed to the impact of moisture, manifesting as the typical X-H hydrogen bond stretching vibration features. Specifically, this change may result from electron transfer between Cl^- as a proton acceptor and O-H as a proton donor, leading to elongation of the O-H bond and triggering its stretching vibration, which increases the absorption intensity and causes an expansion in the wavenumber range. In the 2230-3170 cm^{-1} region, a red shift is observed in the curve after humidity quenching, likely due to moisture-induced changes in molecular coordination, which lead to a structural transition from $[SbCl_6]^{3-}$ to $[SbCl_5]_n^{n-}$. This change causes a red shift in the spectral peak position. At 1600 cm^{-1}, the increase in intensity after humidity quenching may be related to the formation of hydrogen bonds between the nitrogen atom in Mp^+ and water molecules, which alters the C-N stretching vibration and results in an increase in absorption peak intensity.

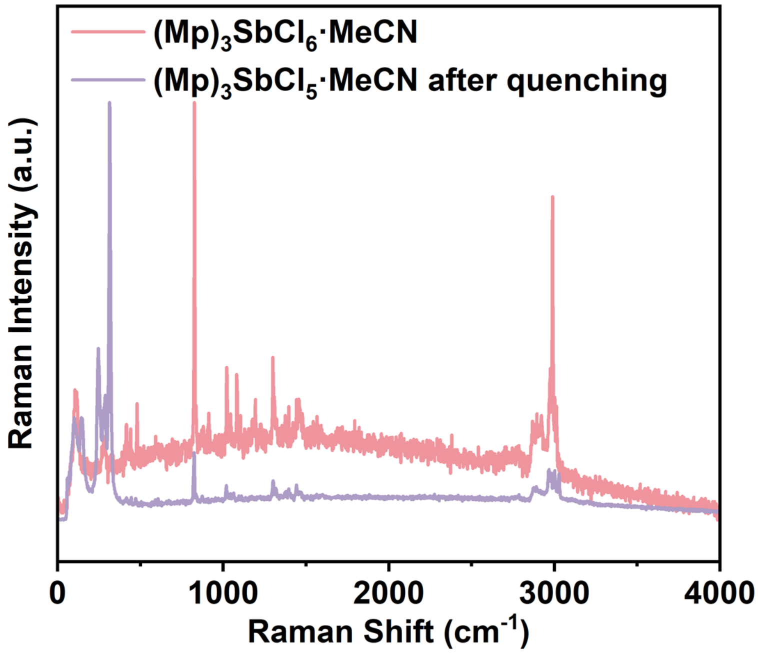

Figure ~~S11~~S12. Raman full spectra of $(Mp)_3SbCl_6{\cdot}MeCN$ and $(Mp)_2SbCl_5$.

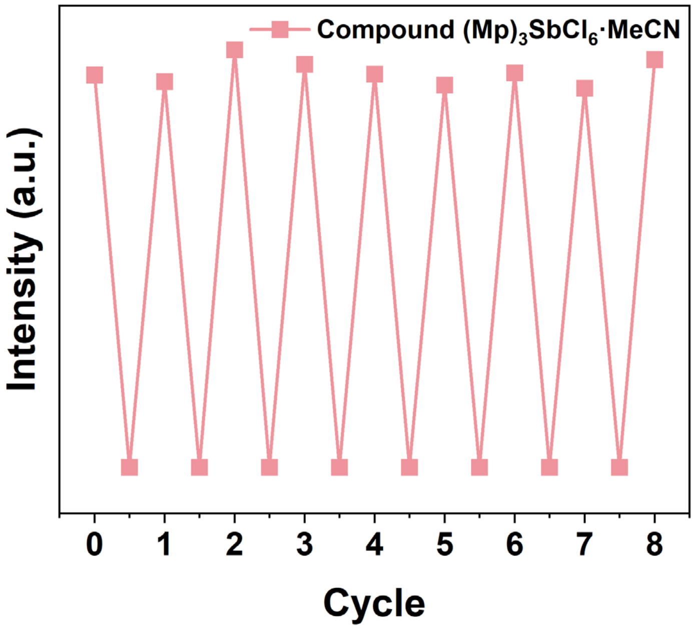

Figure S13. Fluorescence cycling of $(Mp)_3SbCl_6{\cdot}MeCN$.

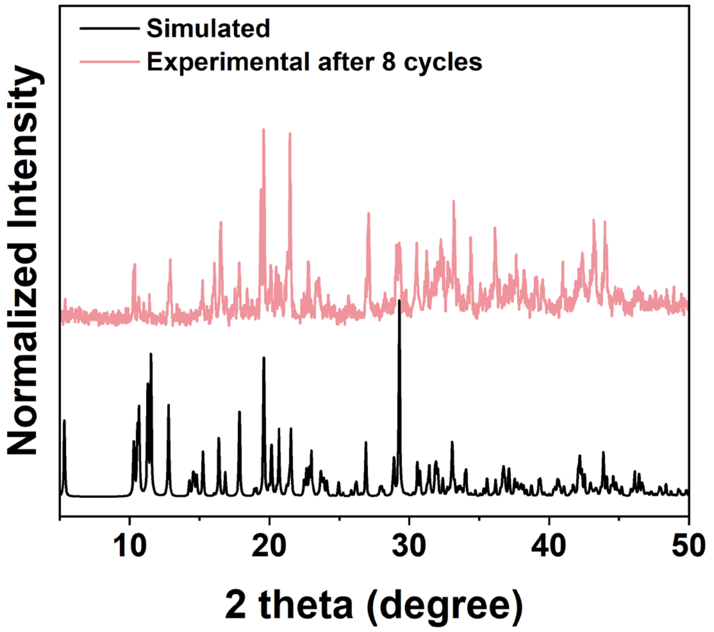

Figure S14. The experimental PXRD pattern of $(Mp)_3SbCl_6{\cdot}MeCN$ after ~~multiple~~ 8 cycles of humidity quenching and fluorescence recovery compared with the simulated one from the single crystal X-ray data of $(Mp)_3SbCl_6{\cdot}MeCN$.
